# Modeling synaptic interactions between mammalian breathing and swallowing central pattern generators

**DOI:** 10.1007/s00422-026-01061-5

**Published:** 2026-09-18

**Authors:** Pavel Tolmachev, Rishi R. Dhingra, Jonathan H. Manton, Mathias Dutschmann

**Affiliations:** 1 Department of Electrical and Electronic Engineering, University of Melbourne, Grattan street 100, Melbourne, VIC 3010 Australia; 2 Case Western Reserve University, 10900 Euclid Ave, Cleveland, OH 44106 USA; 3 Princeton Neuroscience Institute, Princeton University, Washington Road, Princeton, NJ 08540 USA

**Keywords:** Postinspiration, Dorsal swallowing group, Pontine respiratory group, Pre-Bötzinger complex, Digestion, Brainstem, Computational model

## Abstract

Breathing and swallowing are tightly coupled motor behaviors whose execution depends on the coordinated activity of two brainstem central pattern generators (R-CPG, Sw-CPG). We present a mechanistic neural network model that captures this coordination. The model was built by incrementally expanding the synaptic connectivity of R-CPG and Sw-CPG modules, each composed of neuronal population nodes whose dynamics incorporate firing rate adaptation. The rhythmogenic cores of both CPGs are implemented as half-center oscillators operating independently. In agreement with accompanying experimental recordings from an *in situ* perfused brainstem preparation, a simulated 10 s sensory drive to the superior laryngeal nerve (SLN) reliably elicits fictive sequential swallowing bursts accompanied by glottal closure and suppression of inspiratory activity. Short SLN stimuli (100 ms) produce isolated single swallows that reset the phase of the respiratory oscillator. Phase space analysis of the model dynamics offers additional insight into this SLN-evoked respiratory phase resetting. Consistent with prior experimental observations, simulated pontine inhibition reproduces apneusis and abolish swallowing-related glottal closure during sequential swallowing. Systematic perturbation of synaptic weights across specific network pathways identifies vulnerable connections whose weakening recapitulates clinically relevant breathing–swallowing disorders, including aspiration. The model thereby generates mechanistic predictions that may inform the design of therapeutic interventions for conditions characterized by impaired breathing–swallowing coordination.

## Introduction

Swallowing and breathing are two fundamental motor behaviors whose coordination is essential for survival. Unlike breathing, which proceeds autonomously and tolerates only brief interruption before hypoxic injury ensues, swallowing is triggered conditionally—either through sensory afference carried predominantly by the superior laryngeal nerves (Miller and Loizzi 1974; Jafari et al. 2003; Hashimoto et al. 2019; Sang and Goyal 2001) or through descending volitional commands (Mosier and Bereznaya 2001; Ludlow 2015; Wei et al. 2024; Trevizan-Baú et al. 2024). Both air and ingested material share the oropharyngeal passage, and a coordinated sequence of oropharyngeal muscle contractions is required to propel food and liquids from the mouth to the esophagus and onwards to the stomach (Miller 1986; Sejdic et al. 2019; Allen 2012; Jean 2001). A substantial subset of these muscles also participates in the breath-by-breath regulation of upper airway patency (Bartlett 1989; Dutschmann and Paton 2002a; Dutschmann et al. 2014; Bolser et al. 2013; Bautista et al. 2014). Among them, the laryngeal adductors—which draw the vocal folds toward the midline—are active in both contexts. During expiration, graded adduction resists outward airflow and thereby regulates expiratory duration and effort (Harding 1984; Bartlett 2011; Dutschmann and Paton 2002b, a; Dutschmann and Herbert 2006; Dutschmann et al. 2014), while complete adductor-driven glottic occlusion during swallowing shields the subglottic airway from aspiration, a requirement that is particularly stringent during repetitive sequential swallowing (Bolser et al. 2006, 2015; Dutschmann et al. 2014).

The motor programs for respiration and swallowing are produced by anatomically overlapping networks—the respiratory and swallowing central pattern generators (R-CPG, Sw-CPG)—distributed throughout the brainstem (Bianchi and Gestreau 2009). The R-CPG occupies bilateral ponto-medullary columns of respiratory neurons (Alheid et al. 2004), yet accumulating evidence indicates that the characteristic eupneic three-phase motor output—comprising inspiration, postinspiration, and late expiration—depends on synaptic recruitment of the dorsal respiratory group as well (Berger 1977; Hilaire et al. 1990; Dhingra et al. 2019b, a, 2020). This dorsal respiratory group substantially overlaps with the dorsal swallowing group of the Sw-CPG, housed within the nucleus of the solitary tract (NTS) (Jean A. 1979; Kalia and Mesulam 1980; Kessler and Jean 1985; Jean 2001) of the caudal medulla. A second Sw-CPG compartment, the ventral swallowing group, is positioned dorsomedial to the nucleus ambiguus (Kessler and Jean 1985; Ezure et al. 1993; Sugiyama et al. 2011; Fuse et al. 2019; Huff et al. 2023; Toor et al. 2019) and may coincide with the postinspiratory complex (PiCo) (Toor et al. 2019; Pitts et al. 2021; Huff et al. 2023) of the rostral medullary reticular formation, originally described by Anderson et al. (2016) as a distinct excitatory microcircuit generating the postinspiratory phase of breathing. In addition, pontine Kölliker-Fuse neurons have been shown to gate swallow-related sensory transmission and to contribute directly to breathing–swallowing coordination (Bautista and Dutschmann 2014b; Takemura et al. 2022). Taken together, swallowing- and breathing-related neurons are broadly distributed across the ponto-medullary neuraxis. Nevertheless, the specific synaptic pathways linking Sw-CPG and R-CPG populations—and thereby ensuring the precise temporal coupling of the two behaviors—remain incompletely resolved, largely because the functional neuroanatomy of the short- and long-range connections bridging these two CPGs has yet to be fully characterized.

The computational neuroscience of breathing has been dominated for three decades by Hodgkin–Huxley-type models that dissect the synaptic underpinnings of respiratory rhythmogenesis and the three-phase motor pattern (Rybak et al. 1997, 2004a; Smith et al. 2007; Rubin et al. 2009; Molkov et al. 2013, 2014, 2017; Lindsey et al. 2012). Network responses to peripheral sensory challenges—including the Hering–Breuer inflation reflex (Molkov et al. 2013) and hypoxia or hypercapnia (Molkov et al. 2014)—have been captured in such frameworks, yet to our knowledge only a single study has addressed the coupling of two distinct motor CPGs, specifically in the context of respiratory–locomotor entrainment (Potts et al. 2005). Conceptual circuit diagrams for breathing–swallowing coordination have appeared (Bolser et al. 2013; Pitts et al. 2013), but a quantitative, mechanistic computational model has not yet been established.

Here we extend established R-CPG models (Smith et al. 1991; Rybak et al. 2004a; Rubin et al. 2009) by embedding a putative synaptic architecture that enables the coordination of breathing and swallowing within a single unified framework. Both the R-CPG and Sw-CPG are formulated as half-center oscillators (HCOs) cast as Matsuoka neural oscillators (Matsuoka 2011) describing dynamics on a population-level, deliberately emphasizing inter-population connectivity rather than cell-intrinsic biophysics: because the question we address is how the two CPGs are coupled to one another, the model represents the connections between populations explicitly and leaves the conductances within individual neurons unspecified. The model is compared to experimental recordings of SLN-evoked swallowing from an *in situ* perfused brainstem preparation. Under simulated 10 s SLN drive the model reproduces sequential swallowing with simultaneous glottal closure, while a brief 100 ms stimulus reliably triggers a solitary swallow that perturbs the respiratory cycle in a timing-dependent manner. This perturbation is fully described by a phase-resetting curve (PRC) whose profile is in qualitative agreement with prior experimental data (Oku and Dick 1992). Systematic variation of synaptic weights further reveals vulnerable circuit loci whose disruption mirrors clinically relevant breathing–swallowing pathologies.

## Methods

### Experimental data acquisition

All experimental procedures received ethical approval from the Animal Ethics Committee of the Florey Department of Neuroscience & Mental Health, University of Melbourne, Melbourne, Australia (17-077FINMH), and were carried out in full compliance with institutional animal welfare regulations. Juvenile Sprague–Dawley rats of both sexes (postnatal days 17–21) were used in the *in situ* arterially perfused brainstem preparation, following established protocols (Paton 1996; Dutschmann et al. 2000). Animals were deeply anesthetized with isoflurane (2–5%) and, once a surgical plane of anesthesia was confirmed, transected subdiaphragmatically before rapid transfer to an ice-cold bath of artificial cerebrospinal fluid (aCSF; in mM: 125 [$\text {NaCl}$], 3 [KCl], 1.25 [$\text {KH}_2\text {PO}_4$], 1.25 [$\text {MgSO}_4$], 25 [$\text {NaH}\text {CO}_3$], 2.5 [$\text {CaCl}_2$], 10 D-glucose 1.25% Ficoll—an oncotic agent; Sigma–Aldrich, Steinheim, Germany) for decerebration. Following removal of the heart and lungs, the phrenic nerve was dissected free for recording, the descending aorta was exposed for cannulation, the cerebellum was aspirated, and both the vagus and superior laryngeal nerves were isolated for recording and stimulation, respectively.

The brainstem was then transferred to a recording chamber, the aorta cannulated with a double-lumen catheter, and reperfusion commenced with carbogenated (95%/5% pO2/pCO2), warmed ($31^\circ$C) aCSF driven by a peristaltic pump (Watson-Marlow). Phrenic and vagal nerves were drawn onto suction electrodes to capture fictive respiratory and swallowing discharge. Nerve potentials were amplified (400×), band-pass filtered (1–7500 Hz), sampled at 30 kHz through a 16-channel differential headstage (Intan RHD2216), and archived with an Open Ephys acquisition system (Rev. 2; Siegle et al. 2017). Apneustic activity typically resumed within minutes; a bolus of sodium cyanide (NaCN; 0.1 mL, 0.1% w/v) was then injected to provide chemosensory drive that converted the apneustic discharge into a stable eupneic three-phase respiratory motor pattern, after which perfusion flow was fine-tuned to maintain rhythmic stability.

To evoke swallowing, the superior laryngeal nerve (SLN) was stimulated electrically. The current threshold for triggering swallows was first identified using 10 s trains (20 Hz, 0.1 ms pulse width) at amplitudes stepped from 10 to 80 μA; sequential swallowing was then assessed at twice this threshold. To map the SLN phase-resetting curve, single swallows (n=100) were elicited at pseudorandom inter-stimulus intervals of 20–25 s (20 Hz, 250 ms burst duration, 0.1 ms pulse width, 2× threshold current), ensuring coverage of all respiratory cycle phases.

### Experimental data analysis

Signal processing of the vagus and phrenic nerve recordings was carried out in Python using routines from the SciPy library (Virtanen 2020). Each trace was first mean-centred, then bandpass-filtered with a second-order Butterworth filter (signal.butter, passbands: 300 Hz–3 kHz). To prevent phase distortion, every filtering step employed zero-phase forward–backward application (signal.filtfilt). Artifacts from SLN stimulus pulses were removed by linearly interpolating across each stimulus epoch, eliminating the immediate transient deflections without affecting surrounding signal. The absolute value of the resulting signal was taken, and the envelope was further smoothed with a second-order low-pass Butterworth filter (12 Hz cut-off); the processed vagal trace closely resembled a respiratory airflow waveform, and both phrenic and vagal signals became well-suited for downstream analysis. The signals were then decimated by a cumulative factor of 100 through two successive calls to signal.decimate (factor of 10 per step, with anti-aliasing filtering applied at each stage), yielding a final sampling rate of 300 Hz.

The PRC quantifies the dependence of respiratory cycle phase shift on the timing of a brief SLN stimulus within the cycle. To construct it, the continuous recording was segmented into epochs each containing exactly one SLN stimulation event (Fig. 5C). Within each epoch, the phrenic nerve activity (PNA) was additionally low-pass filtered (signal.butter, cut-off frequency = 1 Hz) to isolate the fundamental respiratory oscillation (0.2–1 Hz). The instantaneous phase (protophase) was estimated via the Hilbert transform (Kralemann et al. 2008) following subtraction of the analytic signal mean. The oscillation frequency $\omega _0$ was obtained from a linear fit to the pre-stimulus protophase, and a refined phase ϕ was subsequently computed following Kralemann et al. (2008). Pre- and post-stimulus phase trajectories were each described by linear fits $\phi _b = \omega _0 t + b_1$ and $\phi _a = \omega _0 t + b_2$, with $\omega _0$ held fixed during optimization (optimize.minimize); the phase shift was then computed as $\Delta \phi = (b_1 - b_2)$ (Fig. 5C). The resulting $(\Phi , \Delta \Phi )$ pairs were summarised by a Fourier series fit with a penalty on high harmonics, and a pointwise 95% confidence band for that fit was obtained by resampling the pairs with replacement 500 times and refitting each resample. The transition from advance to delay is defined as the first zero crossing of the fit after the end of the inspiratory burst, the end of the burst being taken as the point where the phase-averaged PNA trace falls below half of its range. The PNA and VNA traces of Fig. 5C show the two linear fits $\phi _b$ and $\phi _a$ defined above, each estimated over the whole pre- and post-stimulus segment of the recording and drawn over a 300 ms window either side of the stimulus.

### Model parameters

Previous studies that modeled R-CPG function incorporated Hodgkin-Huxley style biophysical properties of respiratory neurons (Smith et al. 2007; Rubin et al. 2009; Molkov et al. 2013, 2014, 2017). However, such models are prone to overfitting (Del Negro et al. 2018). Instead of using Hodgkin-Huxley style description, we model neuronal populations with simpler equations underlying the Matsuoka neural oscillator (Matsuoka 2011); which allows the R-CPG and Sw-CPG to function with a reduced parameter set, focusing on their interaction:

$$\begin{aligned} \tau _v \frac{dv}{dt}&= -v - m + (\text {drive} - b)+\text {input} \\ \tau _m \frac{dm}{dt}&= f(v) - m, \end{aligned}$$

where $f(v) = 1/(1+e^{-s v} )$ - activity level of a population, f(v)∈(0,1). In these equations, the dimensionless variable v is analogous to the average membrane potential of a whole neural population. The variable *m* mediates the firing rate (spike frequency) adaptation and acts on a much slower timescale than the variable *v*. To account for the correct timescale of the dynamics and the excitability of the neuronal populations, we use b=0.2, $\tau _v=2$ ms, s=30. Here *b* is a tonic bias on the population input that sets its baseline excitability, i.e. an effective firing threshold, while the -v term is a unit membrane leak, so that *v* relaxes with the membrane time constant $\tau _v$. The parameter $\tau _m$, which controls the timescale of firing rate adaptation, has distinct values for the different neuronal populations (discussed later), and the details are summarized in Table 3. The parameters are chosen manually to match the timescale of respiratory phase durations (inspiration, postinspiration, late expiration) and the number of swallows evoked by the long SLN stimulation during the experimental recordings. The term ‘input’ in the equations incorporates synaptic input from the other nodes of the neuronal populations of the R-CPG or Sw-CPG, as well as the sensory input from the SLN (applicable only to the Sensory Relay neurons, see later). The synaptic input to population j is modeled by the following equation:

$$\begin{aligned} \text {input}_j = \sum _{i \in C}^N w_{ij} f(v_i) +\text {stimulus}_j \end{aligned}$$

**Fig. 1:**
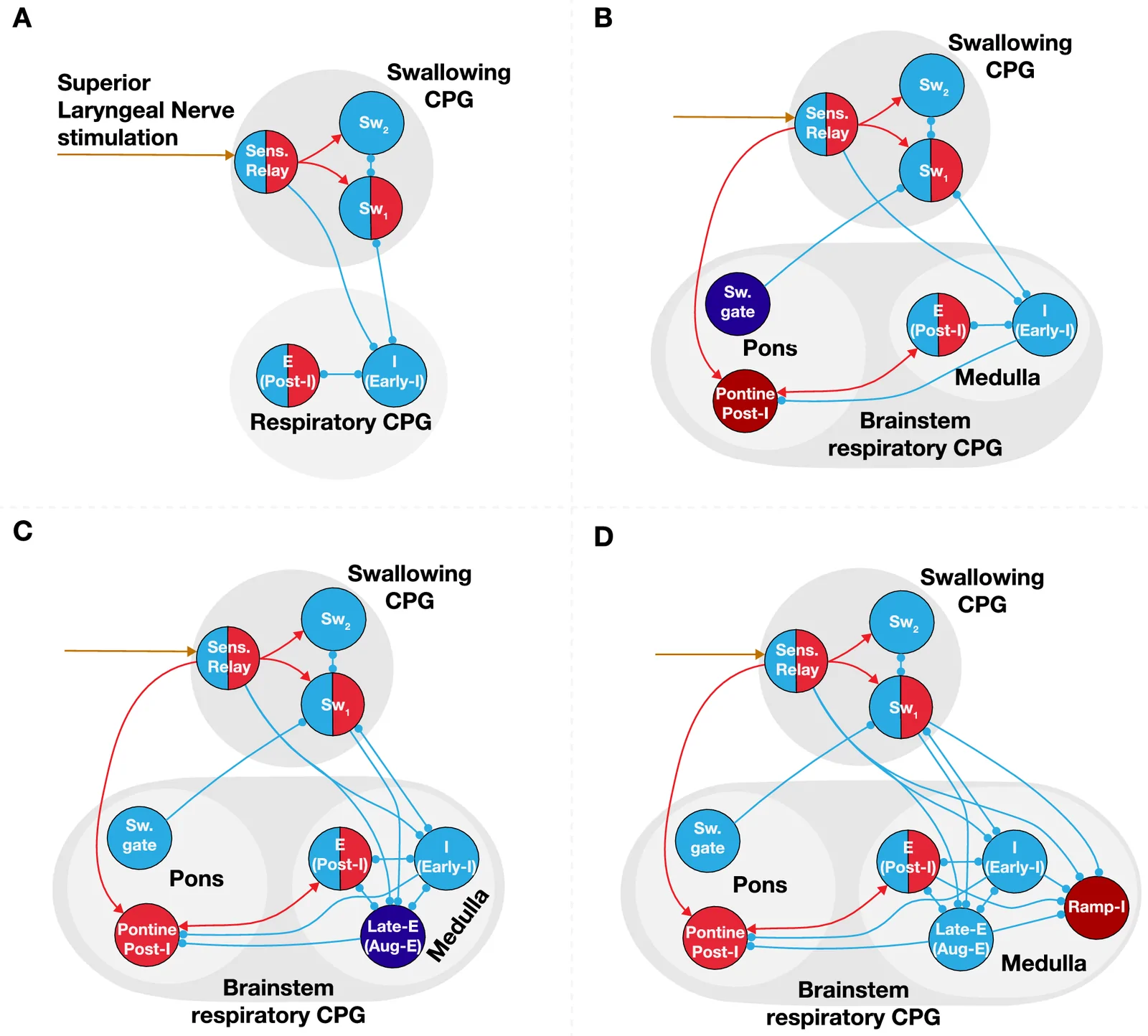
Progression of models for breathing–swallowing circuit coupling. Red and blue nodes represent excitatory and inhibitory populations, with red arrows denoting excitation and circle-capped blue lines—inhibition. For brevity, half-red half-blue populations denote both inhibitory and excitatory subpopulations having the same activity pattern. **A** Starting from the two half-center oscillators for respiratory and swallowing CPGs (R-CPG, Sw-CPG), **B** we add pontine populations: Sw.-gate control, tonically inhibiting Sw-CPG, and Pontine Post-I, driving swallowing-related glottal closure in phase with respiratory Post-I neurons. **C** To obtain the three-phase R-CPG rhythm, we add Late-E population and adjust synaptic strengths. **D** Finally, to capture the ramping inspiratory shape in the phrenic nerve, we introduce Ramp-I population. Newly added populations are highlighted by darker shade

Where *C* is the set of neural nodes projecting to the $j^{th}$ neuron. The stimulus term represents a square pulse input applied to ‘Sensory Relay’ neurons during SLN stimulation. The connectivity parameters $w_{ij}$ between the nodes are manually adjusted to reproduce realistic swallowing and breathing interactions. The gradual development of the model (Fig. 1) allowed for adjusting connectivity parameters step by step, and the connectivity of a simpler model served as a reference point for the connectivity of a subsequent model. The resulting model (Fig. 3) is used for simulation of the phrenic and vagus nerve activities. The baseline level of activity of an isolated neuronal population is controlled by the parameter ‘drive’ which physiologically corresponds to the tonic excitatory drive from the other brain regions.

### Sequential development of the model connectivity

**Fig. 2:**
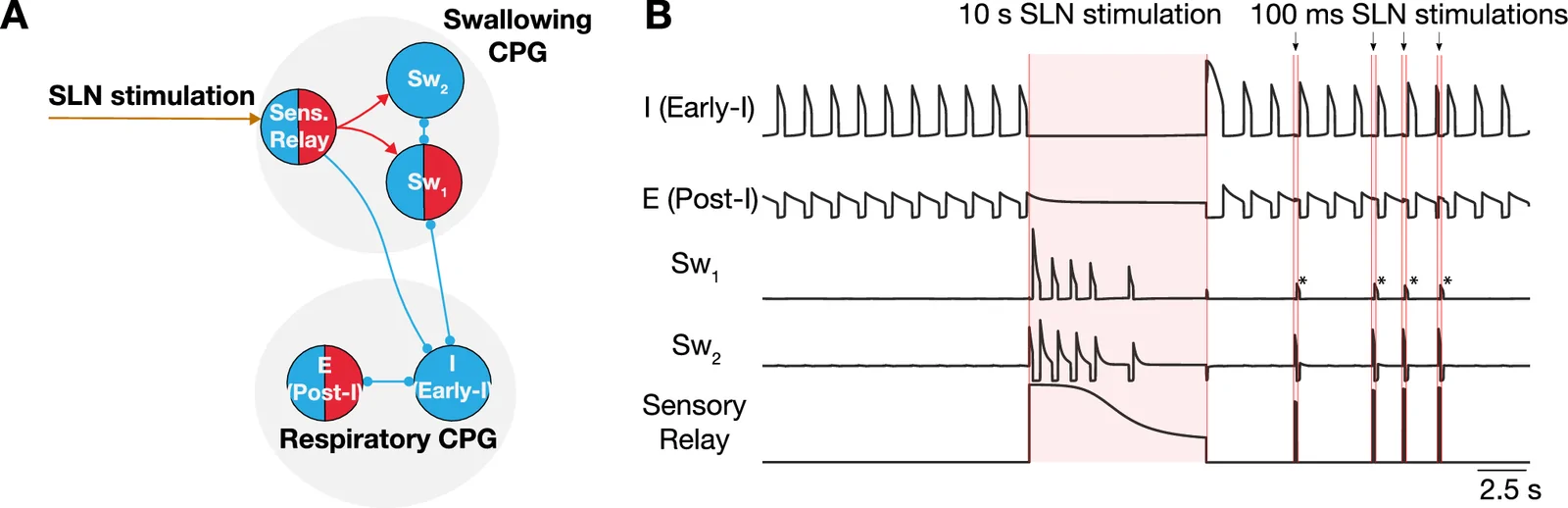
Core model of breathing–swallowing interaction. **A** The respiratory CPG is a half-center oscillator generating inspiration–expiration alternation, while the swallowing CPG is a conditionally active half-center oscillator. **B** During a 10 s sensory stimulus (red shading), the swallowing CPG produces sequential bursts while silencing respiration; a brief stimulus evokes a single swallow (*), interrupting ongoing respiratory activity

Using the equations for the neuronal populations defined above, we developed the network connectivity in 4 successive steps (Fig. 1). The initial model (Model 1; Fig. 1A, Fig. 2) established the basic connectivity: a half-center oscillator in the R-CPG generates biphasic respiratory activity (inspiration (‘I’) and expiration (‘E’)), and a conditionally active half-center oscillator for the Sw-CPG (‘$\text {Sw}_1$’, ‘$\text {Sw}_2$’ populations) produces a sequence of rhythmic swallows in response to a simulated 10 s sensory input from the SLN. The premotor ‘$\text {Sw}_1$’ neurons in Sw-CPG contribute to the vagus nerve discharge. The ‘Sensory Relay’ neurons, whose activity indicates the presence of the sensory stimulus, are comprised of excitatory and inhibitory neuronal populations. The excitatory ‘Sensory Relay’ subpopulation provides drive for the Sw-CPG, while the inhibitory subpopulation suppresses the activity of the inspiratory populations in the R-CPG during ongoing swallowing. In the initial model, a 10 s sensory input from the SLN (Fig. 2) evokes the swallowing related bursts of activity in the vagus nerve output for the larynx that resembles the pharyngeal phase of swallowing (for details see Jean 2001; Bautista and Dutschmann 2014b; Lang 2009). The large value of firing rate adaptation time constant of the ‘Sensory Relay’ neurons is responsible for a slowly declining frequency of the sequential swallows. The simulated SLN stimulation effectively suppresses inspiratory activity during the 10 s period of swallowing. In the next model iteration (Model 2, Fig. 1B), the R-CPG was separated into medullary and pontine compartments to reflect experimental findings that pontine neurons control Sw-CPG gating (Bautista and Dutschmann 2014b). An excitatory postinspiratory population was also added to the pons to account for the tonic postinspiratory activity mediating glottal closure during sequential swallowing (Bautista and Dutschmann 2014b).

**Fig. 3:**
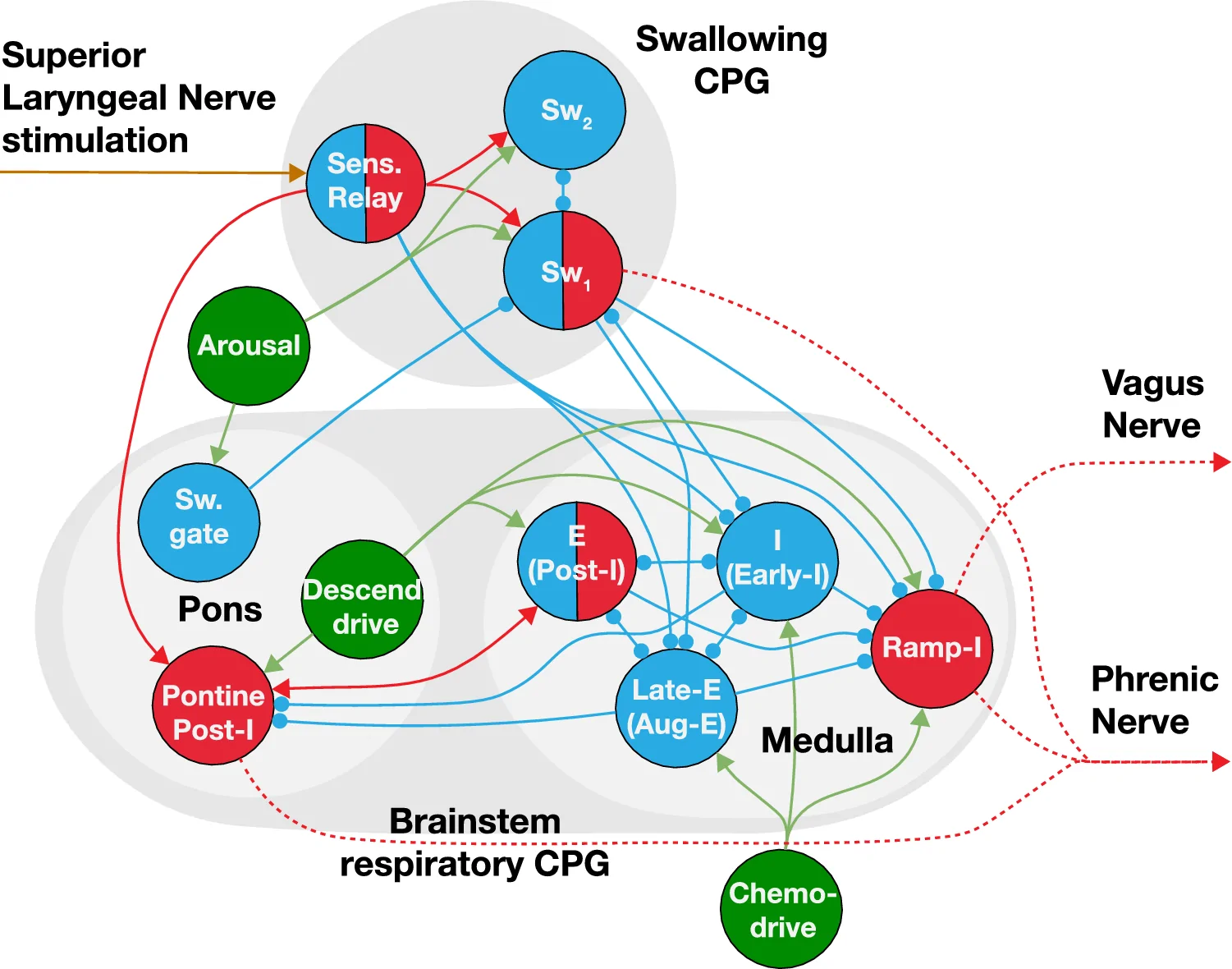
Complete model illustrating the interaction between respiratory and swallowing CPGs with tonic drives. Blue and red circles denote inhibitory and excitatory populations, respectively. Blue circle-capped lines indicate inhibition, red arrows excitation, and double-ended links mutual excitation (red) or inhibition (blue); synaptic strengths differ across connections. Green circles mark tonic drives: (1) arousal drive regulating swallowing neuron excitability, (2) descending pontine drive controlling rCPG core excitability, and (3) chemo-drive adjusting to $O_2$, $CO_2$, and pH. These drives are modeled as constant inputs rather than explicit populations. Superior laryngeal nerve stimulation is shown by brown arrow, and the motor nerve outputs by dashed lines

The next stage of the model evolution (Model 3, Fig. 1C) incorporates an additional expiratory neuronal population to the R-CPG (‘Late-E’) to generate a three-phase respiratory motor pattern, as the hallmark of previous R-CPG model simulations (Smith et al. 1991; Rybak et al. 1997, 2004a; Rubin et al. 2009; Molkov et al. 2013, 2014, 2017). The core of the R-CPG is thus modelled as a ring of three mutually inhibiting populations of inspiratory (‘Insp’), postinspiratory (‘Post-I’) and late-expiratory population (‘Late-E’, active during the second half of the expiration, E2). In the final model (Model 4; Fig. 1D) we incorporate an additional premotor ‘Ramp-I’ population of neurons to account for a eupneic respiratory motor activity that requires a ramping discharge pattern of the simulated phrenic nerve activity (PNA) and vagus nerve activity (VNA) (St-John and Paton 2003; Harris et al. 2017). No intrinsic neural mechanisms have been found to cause a rapid increase in firing rate followed by a sudden stop in activity; therefore, this discharge pattern was hypothesized to result from inhibitory (mainly local) connections (Harris et al. 2017).

In the final model, the outputs of PNA and VNA were simulated as weighted sum of the activities of ‘Ramp-I’, ‘Pontine Post-I’, and ‘$\text {Sw}_1$’ populations. The connectivity parameters between populations are listed in Table 1. We implemented multiple sources of tonic drives for the R-CPG and Sw-CPG neuronal populations in the final model: the ‘Arousal drive’, the ‘Descending pontine drive’ for the medullary respiratory circuit, and the ‘Chemo-drive’ (Fig. 3). In accordance with experimental findings (Sato et al. 2011, 2016), the ‘Arousal drive’ controls the excitability of the Sw-CPG. The ‘Descending pontine drive’ predominantly regulates the excitability of ‘post-I’ neurons in the medullary compartment of the R-CPG (Dutschmann and Herbert 2006; Rybak et al. 2004a, b). Lastly, the ‘Chemo-drive’ projects to ‘late-E’ populations but also provides excitation to the medullary inspiratory neurons. The specific settings for the tonic drives are summarized in Table 2.

**Table 1:** Connectivity description

Node 1 → Node 2	Model 1	Model 2	Model 3	Model 4
SR → $\textrm{Sw}_1$	+0.12	+0.12	+0.12	+0.12
SR → $\textrm{Sw}_2$	+0.07	+0.07	+0.07	+0.07
SR → Pont. Post-I	0.00	+0.03	+0.03	+0.03
SR → I	−0.40	−0.40	−0.40	−0.40
SR → Late-E	0.00	0.00	−0.40	−0.40
SR → Ramp-I	0.00	0.00	0.00	−0.40
$\textrm{Sw}_1 \rightarrow$ $\textrm{Sw}_2$	−0.90	−0.90	−0.90	−0.90
$\textrm{Sw}_1 \rightarrow$ I	−0.80	−0.80	−0.80	−0.80
$\textrm{Sw}_1 \rightarrow$ Ramp-I	0.00	0.00	0.00	−0.80
$\textrm{Sw}_1 \rightarrow$ Late-E	0.00	0.00	−0.20	−0.20
$\textrm{Sw}_2 \rightarrow$ $\textrm{Sw}_1$	−0.65	−0.65	−0.65	−0.65
I → E (Post-I)	−0.30	−0.30	−0.20	−0.20
I → Late-E	0.00	0.00	−1.00	−1.00
I → $\textrm{Sw}_1$	−0.01	−0.01	−0.01	−0.01
I → Pont. Post-I	0.00	−0.35	−0.35	−0.35
I → Ramp-I	0.00	0.00	0.00	−0.22
E (Post-I) → Insp	−0.70	−0.70	−0.50	−0.50
E (Post-I) → Ramp-I	0.00	0.00	0.00	−1.00
E (Post-I) → Late-E	0.00	0.00	−1.30	−1.30
E (Post-I) → Pont. Post-I	0.00	+0.03	+0.03	+0.03
Late-E → E (Post-I)	0.00	0.00	−0.01	−0.01
Late-E → Pont. Post-I	0.00	0.00	−0.65	−0.65
Late-E → I	0.00	0.00	−0.70	−0.70
Late-E → Ramp-I	0.00	0.00	0.00	−0.75
Pont. Post-I → E (Post-I)	0.00	+0.03	+0.03	+0.03
Sw. gate → $\textrm{Sw}_1$	0.00	−0.15	−0.15	−0.15
$\textrm{Sw}_1 \rightarrow$ VN	–	–	–	+0.60
Ramp-I → VN	–	–	–	+0.90
Pont. Post-I → VN	–	–	–	+0.75
Ramp-I → PN	–	–	–	+1.00

SR: Sensory Relay, VN: Vagus nerve, PN: Phrenic nerve

The timescale of firing rate adaptation $\tau _m$ is set to be smaller for the modelled populations in Sw-CPG compared to the neuronal populations of the medullary R-CPG. Such difference in the intrinsic properties is introduced to account for the generation of a physiologically plausible number of swallows during simulated 10 s SLN stimulation. Additionally, we modelled the ‘Sensory Relay’ and the ‘pontine post-I’ populations to have much lower firing rate adaptation than the medullary R-CPG populations, to account for the slower timescale of a decrementing swallowing-related glottal closure activity during 10 s SLN stimulation. The intrinsic properties of the neural groups are summarized in Table 3.

### Modeling of a single swallow in response to short SLN stimulation in the Sw-CPG

In physiological experiments using the perfused brainstem preparation, a short (100–250 ms) stimulation of the SLN evoked a single swallow after the termination of the stimulus, which caused modulation of the respiratory cycle duration (See Results). To account for these experimental findings, ‘Sensory Relay’ neurons excite both ‘$\text {Sw}_1$’ and ‘$\text {Sw}_2$’ populations, which comprise a swallowing half-center oscillator. During eupneic breathing, the ‘$\text {Sw}_2$’ neurons receive more arousal drive than the ‘$\text {Sw}_1$’ neurons, leading to the inhibition of the ‘$\text {Sw}_1$’ premotor population. In our model, a 100 ms SLN stimulation provides additional excitation to both ‘$\text {Sw}_1$’ and ‘$\text {Sw}_2$’ populations; however, the ‘$\text {Sw}_2$’ neurons outcompete the ‘$\text {Sw}_1$’ population, and, at the onset of the stimulus, the ‘$\text {Sw}_2$’ neurons further inhibit the ‘$\text {Sw}_1$’ population. After cessation of the 100 ms SLN stimulation, the ‘$\text {Sw}_1$’ premotor neurons exhibit a post-inhibitory rebound, escaping the ‘$\text {Sw}_2$’ inhibition, thus producing a solitary swallowing motor burst. This mechanism is further analyzed using a phase space analysis (See Results).

## Results

The present computational model of breathing-swallowing interaction depicts each CPG as a half-center oscillator, described by simplified equations adapted from the Matsuoka neural oscillator. In accordance with published data (Fuse et al. 2025), a separate neuron population relays sensory inputs from the SLN to the swallowing network. The respiratory half-center oscillator underlies inspiratory-expiratory phase oscillations, while the swallowing oscillator generates stimulus-dependent sequential pharyngeal swallowing activity (Model 1, Fig. 1A; Fig. 2). Later, we progressively introduce additional neuronal populations (Fig. 1B,C,D) to account for experimental data (both previously published, and obtained presently). The final model (Fig. 3) reproduces the eupneic three-phase motor pattern of respiration characterized by incrementing phrenic nerve activity (PNA) and decrementing postinspiratory activity in the vagal nerve, as shown in previously published models (Rybak et al. 2004a; Smith et al. 2007).

**Fig. 4:**
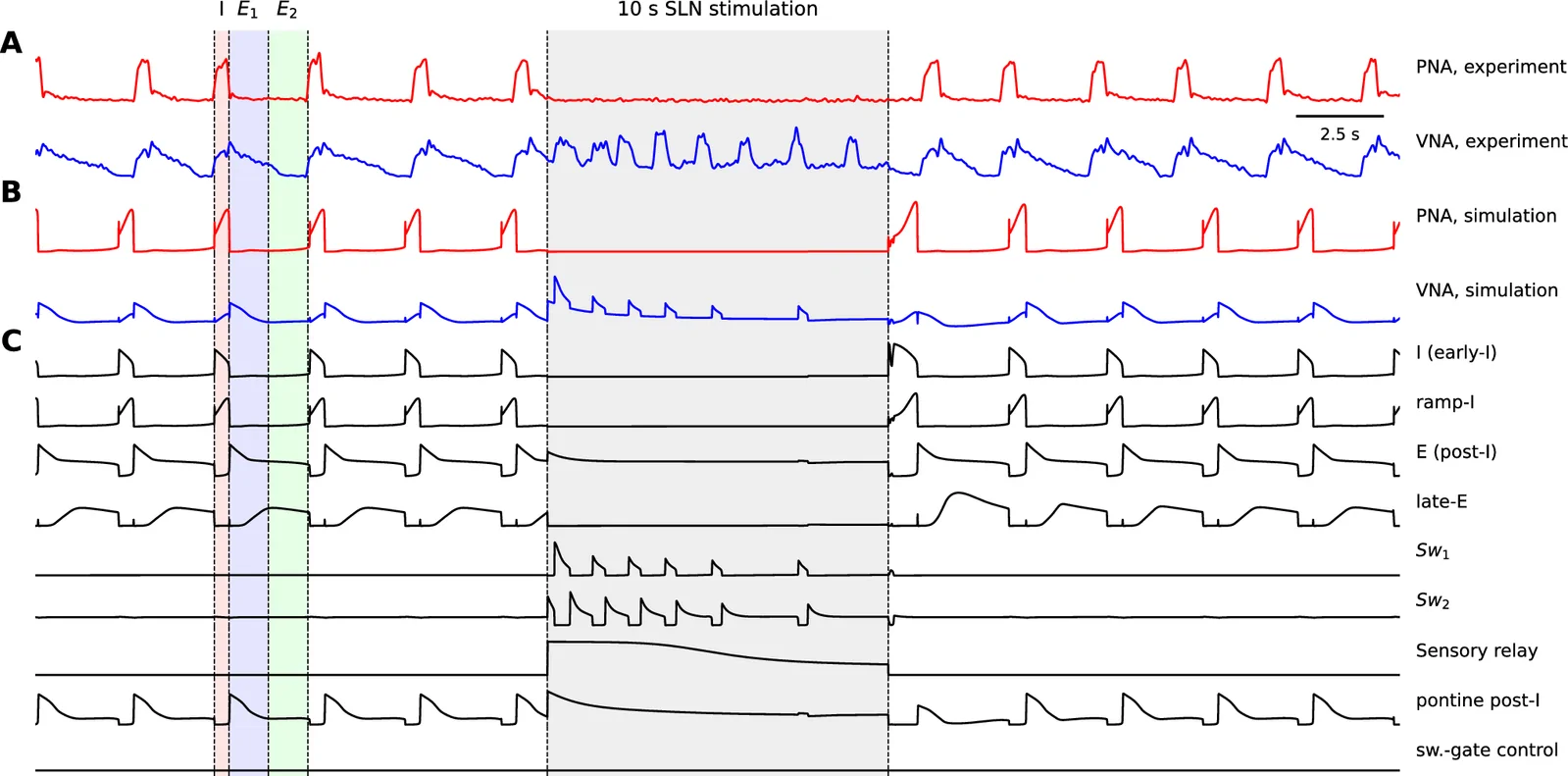
Comparison of the model output with the original recordings. The model reproduces eupneic breathing pattern with distinguished three phases. The comparison of experimental (**A**) and simulated recordings (**B**) from the activities of the phrenic and vagus nerve activities (PNA, VNA) is presented on the four upper traces. (**C**) The rest of the traces show the activities of the individual simulated neuronal populations. The model also qualitatively reproduces the effects of the superior laryngeal nerve (SLN) stimulation. The oscillations in R-CPG are silenced by the ‘Sensory Relay’ neurons, and the respiratory rhythm is locked in the postinspiratory phase. Meanwhile, the sensory neurons activate the Sw-CPG, and the network produces swallowing-related oscillations, with the progressively decreasing frequency of swallows. During the sequential swallowing, the phasically active pontine ‘post-I’ population of neurons in the dorsolateral pons is responsible for the swallowing-related glottal closure. In the model, we assume that the phrenic nerve activity is driven solely by the ‘Ramp-I’ neurons, whereas the vagus nerve activity is simulated as a combination of ‘Pontine Post-I’, ‘Ramp-I’ and ‘$\text {Sw}_1$’ populations with coefficients 0.75, 0.9 and 0.6 correspondingly

**Fig. 5:**
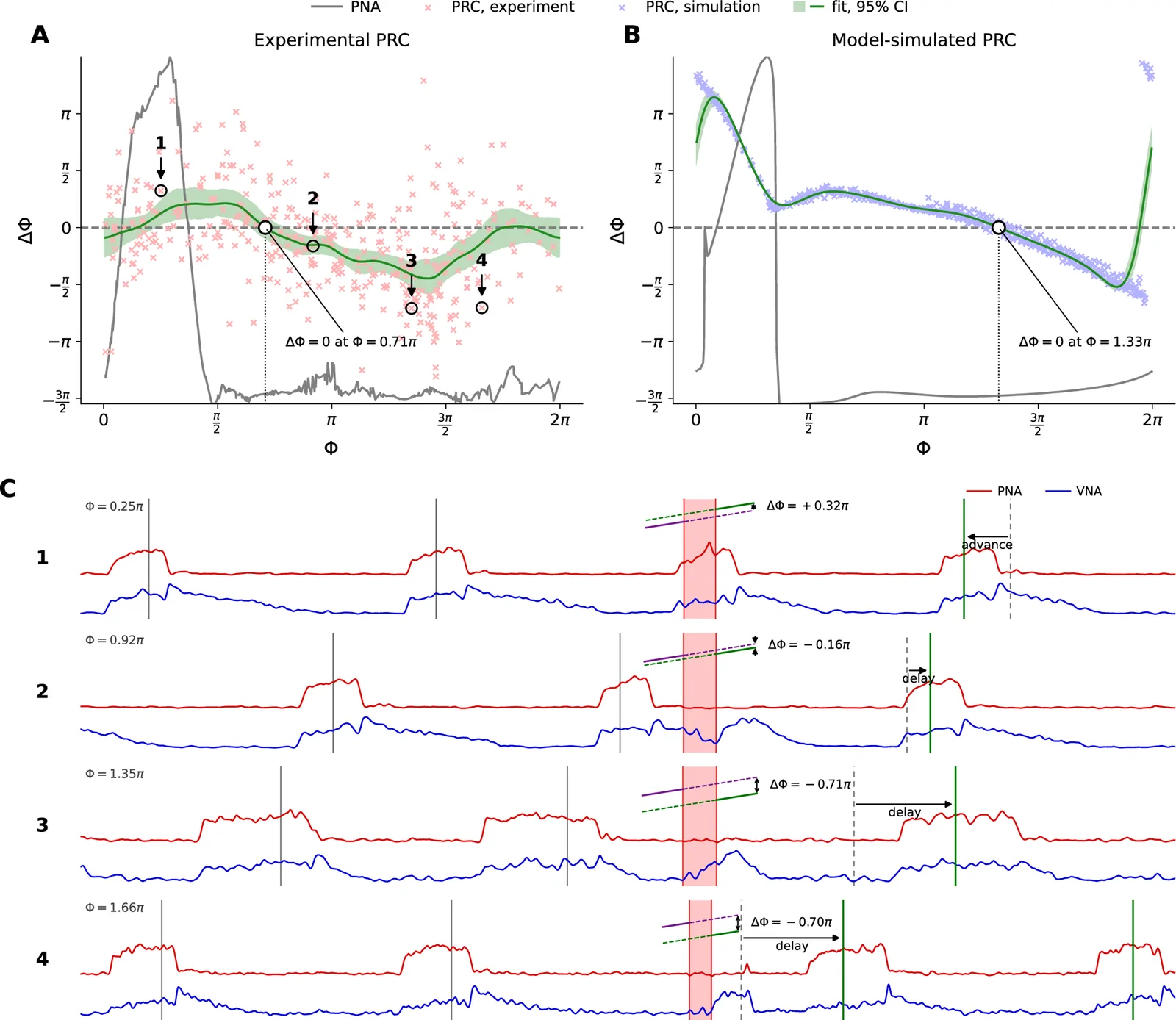
Short stimulation of the superior laryngeal nerve elicits a single swallow, which interferes with the respiratory cycle as characterized by a phase response curve (PRC). The PRC estimated from the experimental data (**A**, 250 ms stimulation, red crosses) is compared with that of the simulated model (**B**, 250 ms stimulation, blue crosses). Each is summarised by a Fourier fit (green) with a pointwise 95% bootstrap confidence band, and the open circle on the zero line marks the transition from advance to delay, labelled with the phase at which it occurs. Numbered circles in **A** mark the four example recordings shown in **C**, each placed at its own measured phase and phase shift. Every panel of **C** shows phrenic (PNA, red) and vagal (VNA, blue) activity with the SLN stimulus shaded, the observed onset of the next inspiration (green) against the onset predicted from the pre-stimulus rhythm (grey dashed), and the phase fitted linearly either side of the stimulus (purple before, green after, dashed where extrapolated), whose vertical separation is the phase shift $\Delta \Phi$. Recording 1 shows a stimulus in the middle of the inspiratory burst, which advances the following cycle; recordings 2–4 fall progressively later in expiration and delay it. See text for details

Simulation of 10 s synaptic input from the SLN forces the two reciprocally inhibiting swallowing populations to compete and oscillate between active (‘$\text {Sw}_1$’) and silent phases (‘$\text {Sw}_2$’) resembling the activity during the pharyngeal phase of swallowing (Hashimoto et al. 2019; Bautista et al. 2014; Kinoshita et al. 2021; Jean 2001). ‘$\text {Sw}_1$’ neurons represent an excitatory premotor subpopulation that drives laryngeal motor output during swallowing, while ‘$\text {Sw}_2$’ interneurons provide no output to upper airway muscles. In parallel, ‘Sensory Relay’ neurons inhibit inspiratory (‘Insp’) and late expiratory (‘Late-E’) neuronal populations of the R-CPG and consequently arrest the three-phase respiratory cycle in the postinspiratory phase (‘Post-I’). In addition, excitatory ‘Sensory Relay’ neurons project to the ‘Pontine Post-I’ neural population, which excites the medullary ‘Post-I’ neurons and drives the vagal motor output to the larynx to mediate the protective swallowing-related glottal closure during sequential swallowing (Bautista and Dutschmann 2014b). Comparison of model performance with experimental data shows that 10 s electrical stimulation of the SLN elicits 5 to 14 swallows, while simulated 10 s SLN input in the model triggers a comparable number of 5–7 swallows (Fig. 4). The model also qualitatively reproduces the experimental data from randomly timed short SLN stimulations in the perfused brainstem preparation: similar to the experimental data, the simulated 100 ms stimulus evokes a single swallowing-related burst, which causes timing-dependent modulation of the respiratory cycle length (Lewis et al. 1990; Oku and Dick 1992, see below; Fig. 5C).

**Table 2:** Excitatory drives to populations

Drive	Value
Chemo drive → I	0.30
Chemo drive → Ramp-I	0.30
Chemo drive → Late-E	0.45
Chemo drive → Pont. Post-I	0.05
Arousal drive → $\textrm{Sw}_1$	${{\textbf {0}}.{\textbf {19}}}^{*}$
Arousal drive → $\textrm{Sw}_2$	0.35
Arousal drive → Sw. gate	0.25
Pontine drive → E (Post-I)	0.35
Pontine drive → I	0.05
Pontine drive → Ramp-I	0.05

${\phantom {a}}^{*}$ For Model 1, the arousal drive to $\textrm{Sw}_1$ is 0.16

**Table 3:** Intrinsic parameters

Population	$\tau _m$ (ms)
I	5000
Ramp-I	5000
E (Post-I)	5000
Late-E	5000
$\textrm{Sw}_1$	1500
$\textrm{Sw}_2$	1500
Sensory Relay	17500
Pont. Post-I	10000
Sw. gate	5000

For all populations: b=0.2, $\tau _v=2$ ms, s=30

### Model validation: phase response curve

Newly acquired and previously reported experimental data obtained in a perfused brainstem preparation of a rat (Paton 1996; Paton et al. 2022) showed that the application of a short train (20 Hz, 250 ms) electrical stimulation of the SLN caused either a decrease or an increase in the respiratory cycle length depending on the timing of the stimulus during the breathing cycle (Fig. 5C). For instance, when the stimulation of the SLN was delivered during the inspiratory phase, the stimulus caused short-latency termination of ongoing phrenic nerve activity, while initiating a solitary swallow (Fig. 5C, trace 1). The subsequent respiratory cycle was shortened due to a decrease in the expiratory phase duration (Horton et al. 2018; Oku and Dick 1992). Short SLN stimulation applied during the late expiratory phase also evoked a solitary swallow; however, in this case the stimulation increased the expiratory phase duration and lengthened the overall respiratory cycle length (Fig. 5C, traces 2–4). The effects of short SLN stimulation are summarized by a phase response curve (PRC), which has become a standard tool for the analysis of oscillatory dynamical systems (Galán et al. 2005; Izhikevich 2007). The experimental and simulated PRCs agree in their two principal features: a phase advance for stimuli delivered during inspiration, and a phase delay that grows through expiration (Fig. 5A,B). They differ, however, in three aspects that are systematic rather than consequences of the low signal-to-noise ratio of the experimental estimate. First, within the inspiratory burst ($\Phi = 0.09\pi$–0.37π) the experimental response varies between advance and delay: 52% of stimulated cycles were shortened and 48% lengthened (n=50). The positive mean phase shift (+0.06π) therefore reflects the larger magnitude of the shortened cycles rather than a systematic advance, the median being only +0.01π; in the model, by contrast, every stimulus delivered during inspiration shortens the cycle (median +0.57π). Second, the transition from advance to delay—the zero crossing of the fitted PRC—occurs at $\Phi = 0.71\pi$ in the experiment, that is 0.34π after the end of inspiration, but at $\Phi = 1.33\pi$, or 0.98π after the end of inspiration, in the model. Third, over late expiration the experimental phase shift returns towards zero: from $\Phi = 1.65\pi$ to the end of the cycle the confidence band of the fit includes zero throughout, so the response there cannot be distinguished from no shift at all. Over the same range the simulated band lies entirely below zero, reaching -0.51π to -0.46π at $\Phi = 1.80\pi$. Additionally, the model’s PRC is discontinuous at the cycle boundary, whereas the experimental one is continuous. We consider the implications of these differences in the Discussion.

### Phase space analysis of the Sw-CPG: transient dynamics of the swallowing half-center oscillator in response to a short SLN stimulation

A short stimulation of the superior laryngeal nerve triggers a swallow that either delays or advances the phase of the respiratory rhythm, depending on the timing of SLN stimulation (Lewis et al. 1990; Oku and Dick 1992). The mechanisms underlying the generation of a short-latency single swallow in response to short SLN stimulation could depend on the ion channel composition (e.g. HCN, BK and SK channels, Molineux et al. (2008)) of SW-CPG neuron populations. Rather than focusing on the kinetics of individual ion channels, we instead modeled the generation of the short-latency swallowing response at a higher level of dynamical abstraction, capturing post-inhibitory rebound of the premotor neurons ($\text {Sw}_1$) using minimal dynamical-system complexity. We implemented an asymmetric tonic excitatory drive (‘Arousal drive’) for the ‘$\text {Sw}_1$’ and ‘$\text {Sw}_2$’ neural populations, along with descending inhibition of the ‘$\text {Sw}_1$’ population from the pontine swallowing gate (‘sw-gate control’). Together, these inputs establish baseline excitation levels of 0.175 for ‘$\text {Sw}_1$’ and 0.35 for ‘$\text {Sw}_2$’. As a result, during SLN stimulation, the ‘$\text {Sw}_1$’ premotor neurons are less excitable than the ‘$\text {Sw}_2$’ population, and the oscillation begins with the motor-silent phase (‘$\text {Sw}_2$’ active). Since ‘$\text {Sw}_1$’ neurons mediate the swallowing-related vagal motor output, this configuration accounts for the physiologically observed delay in swallow initiation following short SLN stimulation (visible in the vagus nerve activity, VNA), as the response initially recruits the ‘$\text {Sw}_2$’ population.

**Fig. 6:**
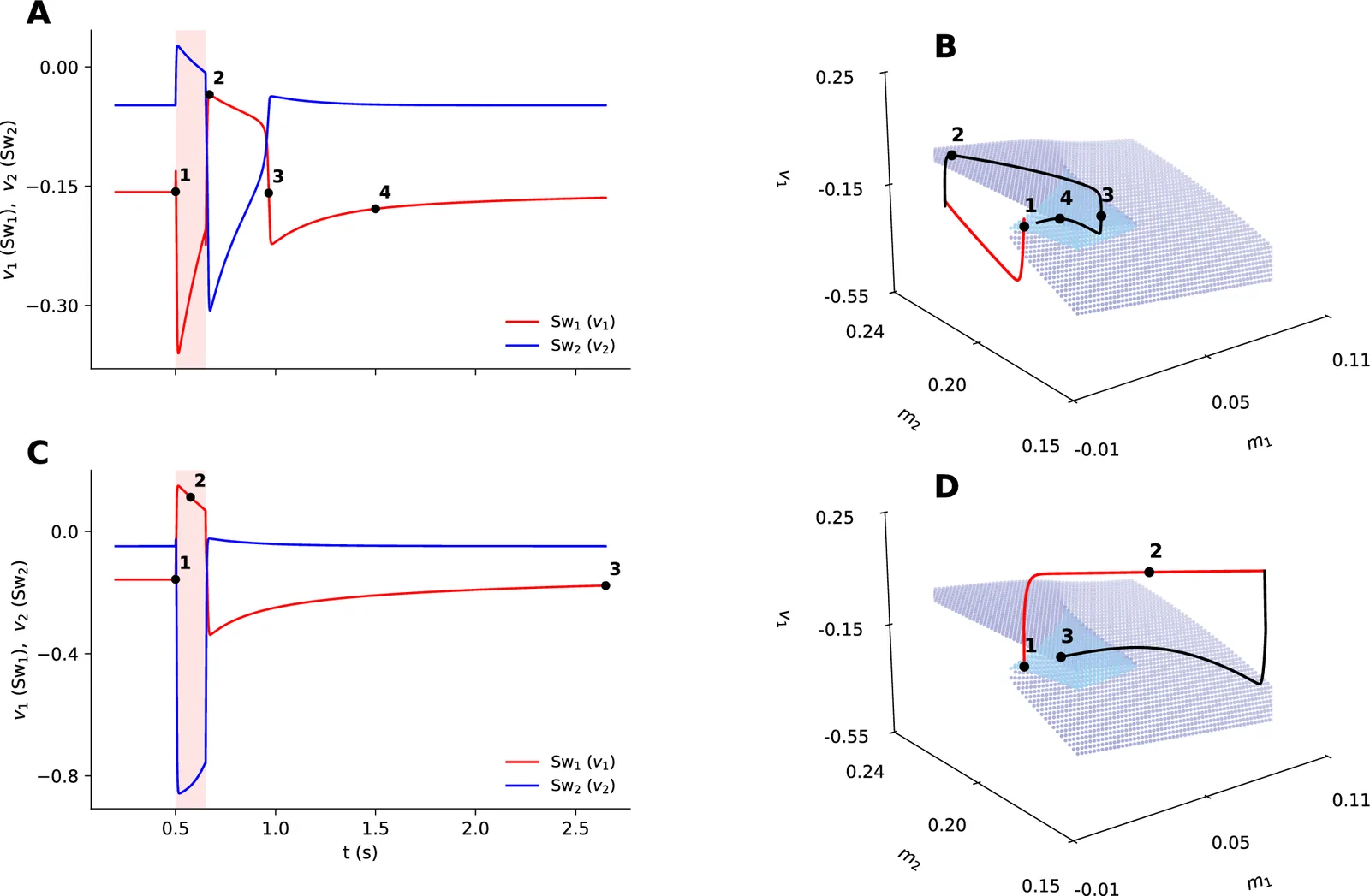
A short 150 ms stimulation of the superior laryngeal nerve (depicted by grey shadings) elicits a response in the Sw-CPG. Here we isolate the swallowing CPG and provide an external stimulus from the ‘Sensory Relay’ neurons to study the mechanisms underlying the generation of the single swallow in the swallowing half-center oscillator. Sub-figures (**A**) and (**C**) depict the simulated membrane-potential traces $v_1$, $v_2$ of the half-center oscillator (index 1 denotes the premotor swallowing ‘$\text {Sw}_1$’ neurons); the numbered points (1–4 in the first scenario, 1–3 in the second) mark the successive stages of the trajectory referenced in the text, while the slow adaptation variables $m_1$, $m_2$ form the horizontal axes of the phase-space panels (**B**, **D**). The swallowing premotor neurons are strongly inhibited in the absence of external input, but are readily excitable. Since the *v*-variables (fast subsystem) evolve on a much faster time scale than the *m*-variables (slow subsystem), the equilibria of the *v*-variables were calculated for each pair of *m*-variables. Taken together, these fixed points form a cusp-like equilibrium surface of the fast subsystem; once the system reaches the stable submanifolds of this surface, its evolution is governed by the slow subsystem. Stable submanifolds are shown in navy blue, unstable equilibria in sky blue. The equilibrium surface is the same in (**B**) and (**D**); the two scenarios differ in the stimulus delivered to each population. The stimulation strengths are (0.12, 0.07) to ‘$\text {Sw}_1$’ (premotor) and ‘$\text {Sw}_2$’ respectively in the first case, and (0.19, 0.07) in the second, where ‘$\text {Sw}_1$’ inhibits ‘$\text {Sw}_2$’ during the stimulus but returns to quiescence after the transient burst. For this isolated analysis the half-center oscillator receives a baseline arousal drive of 0.175 to ‘$\text {Sw}_1$’ and 0.35 to ‘$\text {Sw}_2$’ (the slightly reduced ‘$\text {Sw}_1$’ drive relative to the complete model, 0.19, keeps the resting state close to the cusp; the mechanism is insensitive to this choice)

To see this dynamical mechanism in action, we further analyzed the effect of excitatory inputs from the ‘Sensory Relay’ neuronal population on the Sw-CPG using dynamical system theory. Figure 6 illustrates two different scenarios that discriminate the occurrence of a short-latency single swallow in response to a short SLN stimulation: in the first scenario, a short-latency swallowing response is observed after the stimulation, resembling the swallowing response in the experimental conditions; whereas in the second scenario, the swallowing response is immediate and is concurrent with the stimulus, being unphysiological. Both scenarios differ only in the strengths of the sensory input to the swallowing populations. In the first scenario (Fig. 6A, short-latency single swallow), the strengths of the sensory input to the ‘$\text {Sw}_1$’ and ‘$\text {Sw}_2$’ neurons required a setting of 0.12 and 0.07, respectively. In scenario 2, a small increase of the input strength to the ‘$\text {Sw}_1$’ population up to 0.19 prevents the occurrence of the normal swallowing response to the short SLN stimulation (Fig. 6C, the swallowing response is concurrent with the SLN stimulation).

To study the difference between these scenarios we performed a phase space analysis of the dynamics of the Sw-CPG. The evolution timescales of *v*-variables and *m*-variables differ by three orders of magnitude. Therefore, the whole system can be subdivided into fast and slow subsystems. During the evolution, the $v_1$ and $v_2$ variables are almost functionally dependent, $v_1 \approx f(v_2)$; therefore, one of the fast variables could be eliminated from the analysis, and the dynamics are effectively described in the three-dimensional phase space. For each fixed pair of slow *m*-variables $(m_1,m_2)$, we calculated fixed points of the fast subsystem dynamics $(v_1^*,v_2^*)$, which form a surface. The equilibrium surface in $(m_1,m_2,v_1)$-space forms a cusp (Fig. 6B), a region where the surface folds back on itself so that multiple values of one variable correspond to the same parameter set. This S-shaped structure implies the presence of two stable equilibria on the outer sheets and one unstable equilibrium on the middle sheet. In the global equilibrium (Point 1 in Fig. 6A,B), the system stays on the lower sheet of the equilibrium surface, albeit close to the cusp (lower knee of ‘S’, just before the cusp). The location of the global equilibrium corresponds to the quiescent ‘$\text {Sw}_1$’ neurons, since the value $f(v_1)$ is close to zero, although the system is readily excitable.

In the first scenario, the stimulus drives the system away from the lower sheet of the equilibrium surface into the region of the overhanging upper sheet. The state then jumps ‘vertically’ (along $v_1$ axis) to the upper sheet, from which it gradually slides down until it reaches the boundary of the cusp. While on the upper sheet, the variable $v_1$—representing activity of the premotor $\text {Sw}_1$ neurons—remains elevated, indicating a solitary swallowing response. Once the state reaches the cusp, it abruptly falls back to the lower sheet, corresponding to termination of the solitary swallow (Fig. 6A,B, points 1-2-3-4). In contrast, in the second scenario, the stimulus shifts the system away from the upper sheet, and once the input ceases, the state immediately relaxes back to the lower sheet (where $\text {Sw}_1$ remains quiescent), producing no transient post-stimulus activity (Fig. 6C,D, points 1-2-3). In the model, the swallowing response is therefore determined by the cusp-like shape of the equilibrium surface of the fast subsystem, implementing post-inhibitory rebound of the premotor swallowing population.

### Model validation: silencing of pontine neuronal populations.

**Fig. 7:**
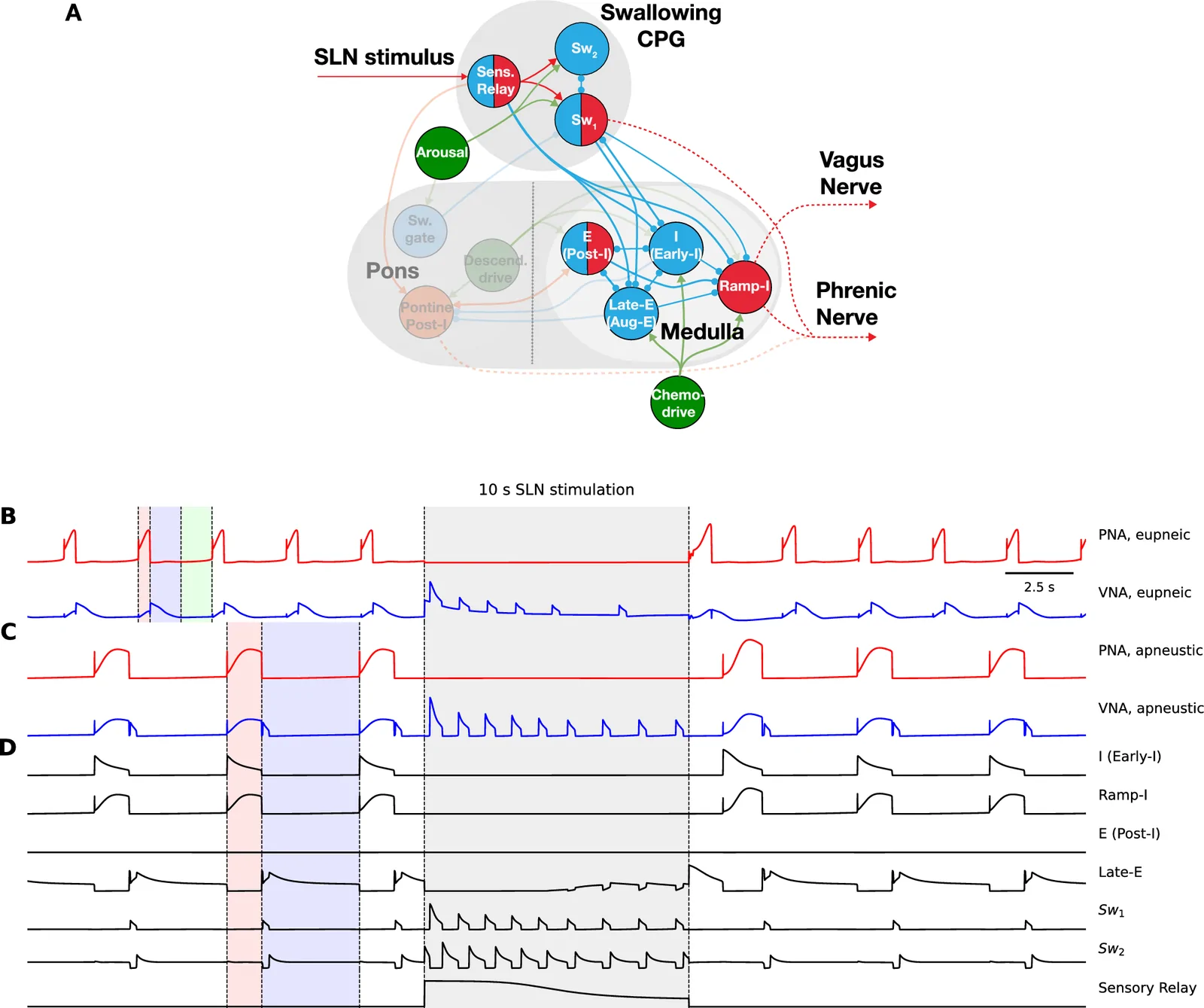
Simulated silencing of the pons influencing the breathing-swallowing coordination. **A** The silenced circuit with all the pontine populations and drives removed. **B** The simulated phrenic and vagus nerve activity (PNA, VNA) of the intact model are compared to the same **C** outputs of the silenced network. **D** The simulated average membrane potential traces of neuronal populations of the silenced circuit. The network demonstrates an apneustic two-phased regime, lacking postinspiratory activity in its output. The 10 s sensory stimulation triggers the sequential swallowing response, although the concomitant swallowing-related glottal closure is absent. The simulated pontine silencing has also alleviated inhibition of premotor swallowing neurons $\text {Sw}_1$, and the latter become active throughout the respiration, resembling the spontaneous activity observed in the experiments

To test whether the proposed ponto-medullary connectivity captures known physiological dependencies, we simulated inhibition of the pontine compartment (Fig. 7). Previously published data have shown that pharmacological inhibition of the pontine compartment of the R-CPG triggered apneusis (prolonged inspiratory phase and absence of postinspiratory motor discharge in VNA, Dutschmann and Herbert (2006)). Inhibition of the pontine populations of the R-CPG had modest effects on SLN-evoked sequential swallowing motor pattern (Takemura et al. 2022) but abolished protective glottal closure during physiologically evoked swallowing (Bautista and Dutschmann 2014b). The latter study also reported that pharmacological inhibition of the pons caused spontaneous swallowing activity in the absence of sensory input. The results of simulated inhibition of the pons are depicted in (Fig. 7B) and the motor output is compared to the simulated eupneic motor activity (Fig. 7A). In line with experimental data, the simulation of pontine inhibition is characterized by an increase in inspiratory phase duration ($T_i$ from 0.44 to 1.30 s) and expiratory phase duration ($T_e$ from 2.37 to 3.71 s) and the absence of postinspiratory discharge in vagal nerve activity. These phase durations are measured per respiratory cycle directly from the inspiratory population activity; because the model is deterministic and settles onto a limit cycle, they are constant across cycles and are therefore reported as single values rather than means. Thus, as shown in previous models (Smith et al. 2007) the respiratory motor pattern is reduced to two phases. This phenomenon is explained by the absence of the descending excitatory drive for the medullary ‘post-I’ population. Thus, the model confirms that pontine drive is necessary for maintaining the three-phase respiratory rhythm.

Apart from its effects on respiration, pontine inhibition is known to alter swallowing behavior (Takemura et al. 2022). To reproduce these experimental observations, we introduced a pontine neuronal population that gates sensory input from the superior laryngeal nerve (SLN) to the swallowing network (‘Sw. gate’ in the pontine compartment; Fig. 1B). We assume that the ‘Sw. gate’ neuron population is intermingled within the pontine respiratory group. In the model, the ‘Sw. gate’ suppresses activation of the swallowing motor population (‘$\text {Sw}_1$’). Consequently, inhibition of the pontine respiratory group also suppresses the ‘Sw. gate’, thereby disinhibiting ‘$\text {Sw}_1$’ and producing spontaneous swallows near the end of the inspiratory phase.

Because the model is deterministic and contains no noise, these spontaneous swallows occur consistently after termination of the apneustic phrenic nerve activity. A qualitatively similar pattern—spontaneous swallowing following apneustic activity—has been observed during autoresuscitation in the *in situ* respiratory network (Bautista et al. 2014), although the experimental timing is much less regular than in the model.

### Model predictions: pathological activity

**Fig. 8:**
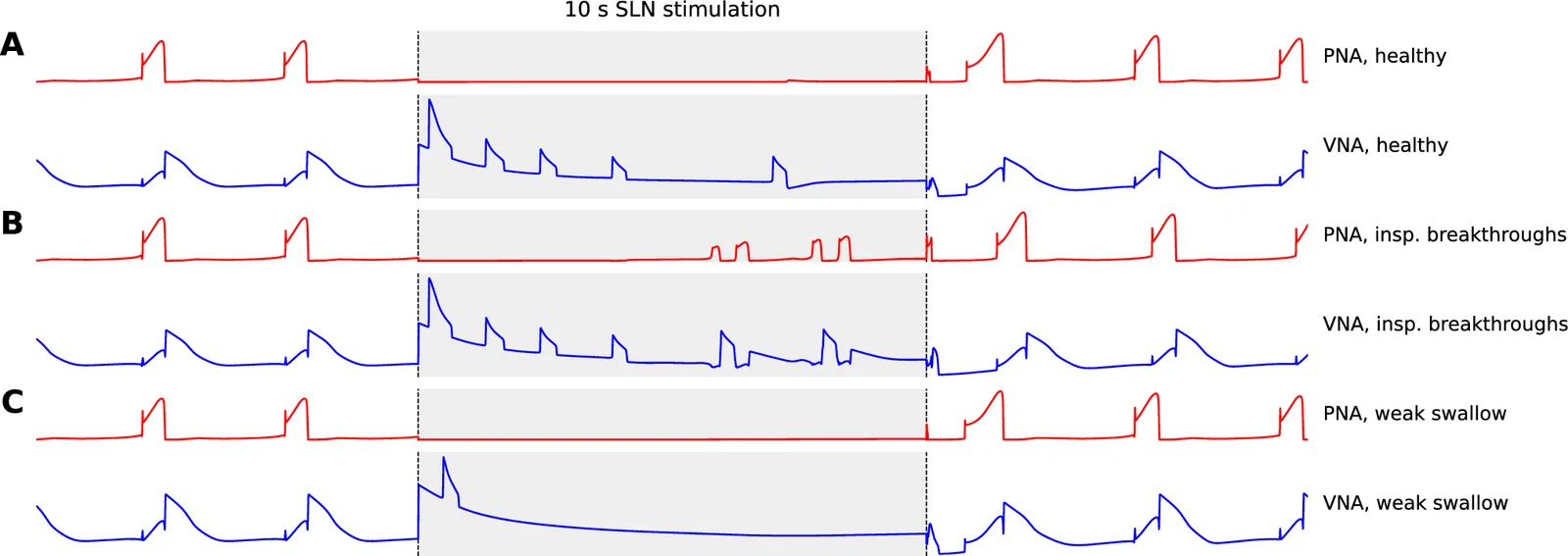
Pathological interactions between breathing and swallowing networks **A** Simulated healthy swallowing response. **B** Simulated pathological activity in the phrenic nerve caused by the weakened sensory inhibition of the inspiratory neurons, both ‘I’ and ‘Ramp-I’. In the simulation, the strength of the sensory inhibition $W_{\text {Sens. Relay} \rightarrow \text {I}}$ and $W_{\text {Sens. Relay} \rightarrow \text {Ramp-I}}$ were both set to -0.22 (vs. -0.4 in the intact network). The simulated inspiratory breakthroughs are biphasic, since the inspiratory neuronal populations are inhibited by the ‘$\text {Sw}_1$’ group, and the latter becomes active during the inspiratory breakthrough transiently inhibiting the pathological inspiration. **C** Weakened and delayed swallowing response in the model of the R-CPG-Sw-CPG network reflected in the response of vagus nerves to the long SLN stimulation. For the simulation, the parameter $W_{\text {Sens. Relay} \rightarrow \text {Sw}_1} = 0.05$ (vs. 0.12 in the intact network). The corresponding traces of the normal swallowing response are shown in the panel for comparison; PNA–phrenic nerve activity; VNA–Vagus nerve activity

In the last part of our study, we performed systematic variations of the synaptic strengths of the network connections. These variations in synaptic strength can predict vulnerable synaptic interactions linked to clinically relevant swallowing disorders. For instance, in our model, inspiratory breakthrough during sequential swallowing occurs when the inhibition from the ‘Sensory Relay’ neurons to inspiratory neurons (‘I’) is decreased below a certain level (Fig. 8A, B, $W_{\text {Sens. Relay} \rightarrow \text {Insp}} = W_{\text {Sens. Relay} \rightarrow \text {Ramp-I}} = -0.22$ instead of -0.4). In our model, these breakthroughs occur toward the end of the evoked sequential swallowing response. Since the inspiratory premotor neurons receive strong inhibition from the ‘$\text {Sw}_1$’ neurons, swallows split the inspiratory phrenic motor discharge into two parts. Physiologically, during the acute phase of inspiration laryngeal abductor activity keeps the glottis open (Dutschmann and Paton 2002a, c); therefore, the modeled activity pattern is likely to cause aspiration (see Discussion for details).

Other clinically relevant pathologies in breathing-swallowing coordination were observed when reducing the strength of the sensory input to the Sw-CPG (Fig. 8A,C, $W_{\text {Sens. Relay} \rightarrow \text {Sw}_1} = 0.05$, whereas the normal value is 0.12). Changes in this synaptic pathway cause a delayed and insufficient swallowing response to the simulated 10 s SLN stimulation. Reduced sensory excitation of the ‘$\text {Sw}_1$’ neuron population results in a greater latency of the first swallow (400 ms vs. 190 ms) and diminishes the number of evoked sequential swallows, resembling the pathological swallowing response in patients with dysphagia (Daniels and Foundas 1997).

### Model predictions: effects of variation of drives, intrinsic properties, and the synaptic connectivity underlying breathing-swallowing interaction

**Fig. 9:**
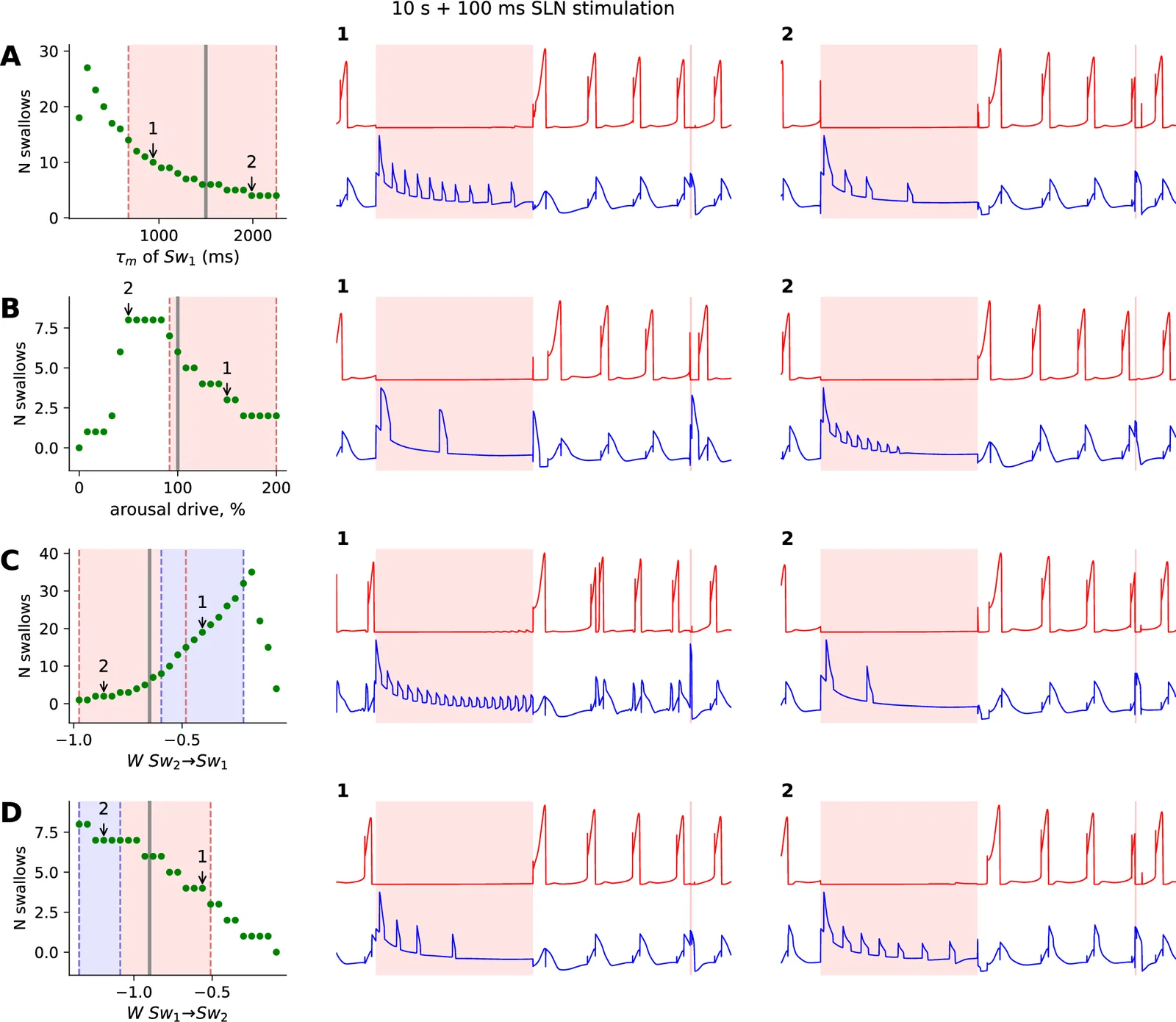
Control of the swallowing: variation of the parameters of the Sw-CPG. To acquire each point on a graph, we simulated the network applying a long stimulation of ‘Sensory Relay’ (Amplitude A=0.45, duration T=10 s) to quantify the sequential swallowing response, and a short stimulation (A=0.45, T=100 ms) – to probe the evoked solitary after-stimulus swallow visible in the simulated phrenic (PNA) and vagus (VNA) nerve responses (example traces 1 and 2, marked by the arrows on the analytics panel). The default value of the parameter is depicted by a grey solid line. Red- and blue-shaded regions highlight the parameter ranges where, respectively, the post-inhibitory-rebound solitary swallow and spontaneous swallows occur. See text for details

We performed multiple numerical perturbations of the model to establish how variation of drive, intrinsic properties, and synaptic connections alter the interaction between the swallowing and the respiratory CPGs. While evaluating the effects of parameter variation, we focused on the number of swallows during the 10 s SLN stimulation (Fig. 9, green dots), occurrence of spontaneous swallows (blue-shaded regions), and the presence of single swallows following a 100 ms SLN stimulation (red-shaded regions).

The variation of firing rate adaptation constant $\tau _m$ of the ‘$\text {Sw}_1$’ population has a drastic effect on the number of produced sequential swallows (Fig. 9A). However, for the lower values ($\tau _m \le 500$ ms), the unphysiologically high number of swallows (reaching up to 27 swallows during a 10 s input simulation) results from non-physiologically short swallow burst durations.

Changes in the arousal drive to the swallowing-related populations revealed that the the relationship between drive strength and number of swallows is not monotonic, reaching a peak of 8 produced swallows at about 80% of the normal value of the drive, while the solitary swallowing response was abolished at the values lower than approximately 90% (Fig. 9B)

Variation of the inhibition from ‘$\text {Sw}_2$’ to ‘$\text {Sw}_1$’ revealed that the number of swallows in the Sw-CPG increases when the inhibition has lower absolute values (from -0.6 to -0.2) reaching as high as 35 swallows. However, the width of these swallows was unphysiologically short (Fig. 9C). Moreover, reducing the inhibition of ‘$\text {Sw}_1$’ by ‘$\text {Sw}_2$’ eventually resulted in persistent pathological swallowing behavior (blue-shaded region).

Perturbing inhibition going the opposite direction—from ‘$\text {Sw}_1$’ to ‘$\text {Sw}_2$’—revealed that the number of swallows in the Sw-CPG declines when the inhibition has lower absolute values (from -1.0 to -0.1) (Fig. 9D), which is expected since the premotor $\text {Sw}_1$ neurons has lesser ability to escape $\text {Sw}_2$ inhibition.

Together, these results indicate that stable and physiologically appropriate breathing–swallowing coordination emerges only within a constrained parameter regime, highlighting the sensitivity of the network to alterations in intrinsic dynamics, tonic drive, and reciprocal inhibition.

## Discussion

Although swallowing disorders are clinically significant in patients with neurodevelopmental and neurodegenerative diseases and are increasingly prevalent in the aging population (Maynard et al. 2020; Sullivan 2008; Gross et al. 2008; Seçil et al. 2016; Easterling and Robbins 2008; Pitts et al. 2008; Finiels et al. 2001; Pitts and Iceman 2023; Niederman and Cilloniz 2022), a sufficiently detailed model of breathing-swallowing interaction has not yet been established (Horton et al. 2018). In the present study, we developed a computational model of the interaction between the respiratory and swallowing CPGs (R-CPG, SW-CPG). To describe the dynamics of these functionally overlapping central pattern generators, we used simplified equations based on the Matsuoka neural oscillator. Accordingly, individual model units (each representing a neural population) omit most biophysical detail and retain only firing rate adaptation as the minimal mechanism for rhythmogenesis. This simplification allowed us to focus on the synaptic connectivity between neuronal populations that coordinate breathing and swallowing. The R-CPG model builds upon previously published models (Rybak et al. 2004a; Smith et al. 2007; Molkov et al. 2013, 2017) that reproduce the three-phase eupneic breathing motor pattern. Extending these models, the present Sw-CPG/R-CPG network additionally generates both solitary and sequential swallowing responses. The modeled synaptic interactions qualitatively reproduce physiologically observed breathing-swallowing coordination. Computational experiments involving circuit ablations and systematic variation of synaptic strengths reveal potential mechanisms of breathing-swallowing miscoordination. Overall, the model provides a mechanistic framework that generates testable predictions and advances understanding of the network basis of swallowing disorders.

### Network dynamics and biophysical properties of neurons

The current model of the interaction between the SW-CPG and R-CPG is strongly based on previous computational models describing the mammalian respiratory circuit (Molkov et al. 2017). Previous R-CPG models utilized conductance-based formulations featuring Hodgkin–Huxley–type neurons (Smith et al. 2007; Molkov et al. 2013; Rybak et al. 2004a; Rubin et al. 2009; Molkov et al. 2014). Following the discovery of rhythm-generating neuron populations with intrinsic bursting properties in the Pre-Bötzinger complex (Smith et al. 1991), modeling efforts increasingly focused on reproducing rhythmogenic properties through intrinsic neuronal dynamics (Butera et al. 1999; Ptak et al. 2005). Although Hodgkin–Huxley–based models incorporating ionic currents are biophysically detailed, in practice many intrinsic parameters must be hypothesized or extrapolated rather than directly determined (Del Negro et al. 2018). This can lead to overparameterization of the system and obscure the underlying network mechanisms. Moreover, studies have demonstrated that qualitatively similar discharge patterns and dynamical behaviors can emerge from neurons with markedly different ion channel compositions in rhythmic neural circuits (Schulz et al. 2006; Marder and Bucher 2007; Goaillard et al. 2009; Marder et al. 2015). More recent computational work further suggests that the contribution of intrinsically bursting neurons in the intact respiratory network may be masked by mutual synaptic inhibition required for the formation of the three-phase respiratory motor pattern (Rubin and Smith 2019; Wang and Rubin 2020; Tolmachev et al. 2018; Guerrier et al. 2015). Taken together, these considerations have not displaced intrinsic conductances in favour of synaptic interactions so much as established that the two are interdependent sources of network dynamics. That degeneracy implies that a given network output can be produced by many different combinations of intrinsic and synaptic properties (Golowasch 2014), whose relative contributions shift with the state of the network rather than one superseding the other (Ramirez and Baertsch 2018; Del Negro et al. 2018). Consistent with this, intrinsic bursting in the Pre-Bötzinger complex appears to be conditional—modulated by network state and drive—rather than strictly required for rhythmogenesis (Del Negro et al. 2018; Rubin and Smith 2019; Ramirez and Baertsch 2018).

In this work, the emphasis on synaptic architecture is justified by the scope of the question we asked. Our object of study is the coordination between two central pattern generators. This is a network-level phenomenon, and it is naturally addressed by making inter-population connectivity explicit. Therefore, rather than adopting conductance-based formulations, our approach describes neural populations using simplified equations that capture essential dynamical features, thereby enabling a focused analysis of interactions within the respiratory motor circuit that generate the three-phase eupneic respiratory motor pattern (Molkov et al. 2017; Tolmachev et al. 2018). In accordance with these studies, we omitted intrinsic bursting properties of PreBötC neurons in the R-CPG model. Similarly, we did not include specific biophysical properties in the SW-CPG due to the absence of detailed experimental data. Instead, we modeled the SW-CPG using a half-center oscillator motif with firing rate adaptation. These assumptions are supported by experimental findings showing that local $\text {GABA}_{\text {A}}$ receptor blockade and consequent disruption of synaptic inhibition in the dorsal swallowing group abolish sequential swallow burst generation (Bautista and Dutschmann 2014b). The biological validity of the model is further supported by its ability to reproduce a decrementing swallow motor burst frequency during a 10 s SLN stimulation, consistent with experimental observations (Fuse et al. 2019, 2025; Takemura et al. 2022; Hashimoto et al. 2019; Kinoshita et al. 2021). Together, these results indicate that the essential dynamics of breathing–swallowing coordination can be captured through interactions among inhibitory neuronal populations, without specifying detailed intrinsic rhythmogenic properties of individual neurons. This demonstrates that the connectivity is sufficient to account for the coordination at this level of description; however, it does not establish that intrinsic properties are dispensable in the biological network. A corollary of this network focus is that the model cannot, by construction, predict the effects of pharmacological or genetic manipulations targeting specific channels or receptors. Doing so would require channel- and receptor-resolved data in identified populations (transcriptomic profiling of ion-channel and receptor expression, voltage-clamp dissection of the relevant intrinsic currents, per-population conductance profiling, and dynamic-clamp validation), which would let detailed models predict how targeting specific receptors in specific populations alters the integrated behavior and thereby guide interventions for breathing–swallowing disorders. The network-level model presented here is a deliberately modest but necessary first step toward that goal.

However, even when attention is restricted to network connectivity, substantial challenges remain. The available experimental data on connectivity are incomplete, and the distribution of neuronal cell types across compartments—for instance, as reported by Molkov et al. (2025)—is not well captured by a reduced circuit of the present kind. A further challenge is that the connection weights our model posits are underconstrained: reproducing the observed motor patterns and a handful of experimental observations does not uniquely determine them. Recent conductance-inference techniques, which decompose single-neuron recordings in rhythmically active circuits into respiratory cycle-phase-dependent excitatory and inhibitory conductances (Molkov et al. 2025, 2026), offer a route to estimate these weights directly from data and, applied during fictive swallowing, to test the specific breathing–swallowing connections we predict (e.g. the SLN-driven inhibition of inspiratory premotor neurons or the pontine gating of the swallowing circuit).

### Postinhibitory rebound dynamics—a putative mechanism for solitary swallow generation

Interneurons in CPGs and associated half-center oscillator models often display post-inhibitory rebound activity (Hooper 2001; Bucher et al. 2015; Nagornov et al. 2016; Jean 2001). Postinspiratory rebound bursting has been linked to specific ion channels, such as slow synaptic NMDA-receptor–mediated excitation (Jean 2001; Tell and Jean 1993), transient outward potassium current $I_{\text {KA}}$ (Jean 2001; Tell and Jean 1991), or low-threshold–activated calcium channels $I_T(\text {Ca})$ (Tell and Bradley 1994; Champagnat et al. 1986; Jean and Dallaporta 2006). The latter have been indeed reported to mediate post-inhibitory rebound in swallowing-related neurons in the nucleus of the solitary tract (Tell and Bradley 1994; Champagnat et al. 1986; Jean and Dallaporta 2006). Here, we abstracted away from these specific ion channels while demonstrating that post-inhibitory rebound dynamics may underlie the short latency of single-burst swallowing activity (Fig. 6). In our model, the generation of a single short-latency swallow is attributed to the dynamics of the half-center oscillator as a whole, operating in a quasi-bistable regime. This transient excursion between stable states provides the dynamical substrate for both solitary swallow generation and the associated respiratory phase resetting.

Finally, short SLN stimulation reliably evokes a single swallow burst that causes a respiratory cycle phase shift. In accordance with our experimental data and previous work (Oku and Dick 1992), SLN stimulation terminates inspiration and shortens the expiratory interval, whereas stimulation during the later stage of expiration prolongs the expiratory interval. In our model, the swallow-evoked phase resetting is explained by post-inhibitory rebound of swallowing premotor neurons and inhibition of inspiratory populations by these premotor neurons (Horton et al. 2018; Saito et al. 2002, 2003).

The three differences about PRC reported in the Results indicate what the present model does not capture. The variable phase-advance or delay during inspiration cannot be reproduced by a deterministic model: in the model, every stimulus evokes a swallow, the swallow inhibits and terminates inspiration, and post-inhibitory rebound then restarts it, so the cycle is substantially advanced on every trial. In the preparation the outcome varies between trials, in part because a stimulus does not always evoke a swallow; Lewis et al. (1990) likewise found that superior laryngeal nerve stimulation terminates inspiration on some trials and only briefly suppresses it on others. The late transition from advance to delay may have the same origin, since the probability of evoking a swallow is not constant across the respiratory cycle in the intact animal, so the measured curve averages trials with and without a swallow. A possible explanation for the stimulus-insensitive zone in late expiration could be that inspiratory neurons become progressively harder to suppress, through intrinsic bursting properties that the model does not include. Overall, the analysis presented here outlines experimentally observed phenomena that could serve as new benchmarks for model validation. A stochastic model, in which swallow initiation is probabilistic and depends on respiratory phase, would be the natural next step.

### Mediation of the swallowing-related glottal closure reflex and the swallowing breath-hold

A key feature of swallowing–breathing coordination is the protective breath-hold and glottal closure during sequential swallowing (Harding 1984; Bautista et al. 2014; Dutschmann et al. 2014; Pitts and Iceman 2023; Sun et al. 2011), particularly during liquid ingestion. Multiple studies have established that the Kölliker–Fuse area (KF) in the dorsolateral pons gates postinspiratory activity in the efferent vagus, which modulates the pattern of expiratory airflow via partial laryngeal adduction during the early expiratory (postinspiratory) phase (Dutschmann et al. 2014; Browaldh et al. 2016). It has been suggested that KF neurons driving laryngeal adductor muscle activity during breathing also mediate glottal closure during sequential swallowing, since pharmacological inhibition of the KF eliminates both breathing- and swallowing-related vagal postinspiratory discharge (Bautista and Dutschmann 2014b). Other studies have shown that swallowing-related glottal closure may also originate from postinspiratory neurons in the BötC (Sun et al. 2011) or from the postinspiratory complex in the rostral medulla oblongata (Toor et al. 2019; Huff et al. 2023). Despite the likelihood that, in vivo, swallowing motor activity involves an anatomically distributed network (Jean 2001; Sugiyama et al. 2011; Sun et al. 2011; Bautista and Dutschmann 2014b), we simplified the model by introducing pontine post-I neurons as the primary drivers of glottal closure during swallowing. Additional studies have shown that glottal closure and inspiratory inhibition may be governed by separate synaptic pathways, in which sensory inputs from the SLN inhibit inspiratory premotor neurons (Hiss et al. 2003; Bautista et al. 2014; Sun et al. 2011). We incorporated these findings by introducing a subpopulation of inhibitory sensory relay neurons that project to inspiratory premotor neurons and interneurons.

### Pathological breathing-swallowing interaction and neurologic dysphagia

Systematic variation of synaptic strengths in specific network connections revealed potential sources of clinically relevant vulnerabilities in the interactions between the SW- and R-CPG. Dysphagia and swallowing disorders can be classified according to the timing of the swallow motor act within the respiratory cycle. Swallowing occurring during the inspiratory phase is associated with an increased risk of aspiration (Yagi et al. 2017; McCarty and Chao 2021), as is delayed initiation of swallows during expiration (Yagi et al. 2017; McCarty and Chao 2021). The latter can cause choking, airway obstruction, and aspiration due to desynchronization between the delayed swallow and protective glottal closure. Variation of specific synaptic pathway strengths indicates that pathological swallowing during inspiration may arise either from decreased inhibition between the premotor swallowing neuron population and the medullary pre-inspiratory neuron population, or from reduced synaptic drive from sensory relay neurons responsible for suppressing inspiratory interneurons and premotor neurons. A delay in the swallowing response due to increased swallowing latency may result from reduced excitation of swallowing premotor neurons or insufficient inhibition of these neurons by pontine populations. The model further suggests that a pathological increase in drive to sensory NTS neurons projecting to the medulla could also contribute to disordered breathing–swallowing coordination. Thus, the model links distinct clinical patterns of dysphagia to specific synaptic mechanisms within the breathing–swallowing network.

## Conclusion

In the present study, we introduce a first computational model of a putative network connectome underlying the coordination of swallowing and breathing. The model integrates and supports several key experimental findings. It represents a first step toward predicting network dysfunction underlying dysphagia and aspiration. These conditions frequently lead to fatal pneumonia in the elderly (Finiels et al. 2001; Easterling and Robbins 2008) and in patients with stroke (Leslie et al. 2002), chronic obstructive pulmonary disease (Gross et al. 2009; Nagami et al. 2017), Parkinson’s disease (Gross et al. 2008), and Alzheimer’s disease (Seçil et al. 2016). Some of the specific model predictions centered on ponto-medullary synaptic interactions during swallowing–breathing coordination may also align with findings from transgenic mouse models of tauopathy that suggest severe neurodegeneration in the NTS and KF circuits (Rüb et al. 2001; Dutschmann et al. 2010; Menuet et al. 2011), both of which are critical components of the present computational framework. Although the present model represents only an initial step, it provides a framework for iteratively refining network connectivity hypotheses, highlighting circuit-level synaptic architecture as an important determinant of pathological breathing–swallowing coordination.

## Data Availability

The experimental recordings of phrenic and vagal nerve activity during short and long SLN stimulation are publicly available on Zenodo at: https://doi.org/10.5281/zenodo.18750367

## Code Availability

The code for modeling, PRC extraction, and experimental data processing is available on GitHub at https://github.com/ptolmachev/SimplifiedModel_rCPG_swCPG_interaction.

## References

[CR1] Anderson TM, Garcia AJ, Baertsch NA, Pollak J, Bloom JC, Wei AD, Rai KG, Ramirez J-M (2016) A novel excitatory network for the control of breathing. Nature 536(7614):76–80 27462817 10.1038/nature18944 PMC5479418

[CR2] Allen JE (2012) Deglutition, swallowing, and airway protection: physiology and pathophysiology. Gastroesophageal Reflux Lung 25:1–22

[CR3] Alheid GF, Milsom WK, McCrimmon DR (2004) Pontine influences on breathing: an overview. Respiratory Physiol Neurobiol 143(2–3):105–114 10.1016/j.resp.2004.06.016 15519548

[CR4] Browaldh N, Bautista TG, Dutschmann M, Berkowitz RG (2016) The kölliker-fuse nucleus: a review of animal studies and the implications for cranial nerve function in humans. Eur Arch Otorhinolaryngol 273(11):3505–3510 26688431 10.1007/s00405-015-3861-9

[CR5] Bautista TG, Dutschmann M (2014) Ponto-medullary nuclei involved in the generation of sequential pharyngeal swallowing and concomitant protective laryngeal adduction in situ. J Physiol 592(12):2605–2623 24639482 10.1113/jphysiol.2014.272468 PMC4080941

[CR6] Berger AJ (1977) Dorsal respiratory group neurons in the medulla of cat: spinal projections, responses to lung inflation and superior laryngeal nerve stimulation. Brain Res 135(2):231–254 922474 10.1016/0006-8993(77)91028-9

[CR7] Bautista TG, Fong AY, Dutschmann M (2014) Spontaneous swallowing occurs during autoresuscitation in the in situ brainstem preparation of rat. Respiratory Physiol Neurobiol 202:35–43 10.1016/j.resp.2014.07.015 25086277

[CR8] Bianchi AL, Gestreau C (2009) The brainstem respiratory network: an overview of a half century of research. Respiratory Physiol Neurobiol 168(1–2):4–12 10.1016/j.resp.2009.04.019 19406252

[CR9] Bolser DC, Gestreau C, Morris KF, Davenport PW, Pitts TE (2013) Central neural circuits for coordination of swallowing, breathing, and coughing: predictions from computational modeling and simulation. Otolaryngol Clin North Am 46(6):957–964 24262953 10.1016/j.otc.2013.09.013 PMC3863908

[CR10] Bucher D, Haspel G, Golowasch J, Nadim F (2015) Central pattern generators eLS. pp 1–12

[CR11] Bartlett D Jr (1989) Respiratory functions of the larynx. Physiol Rev 69(1):33–57 2643125 10.1152/physrev.1989.69.1.33

[CR12] Bartlett D Jr (2011) Upper airway motor systems. Compr Physiol. 10.1002/cphy.cp030208

[CR13] Butera RJ Jr, Rinzel J, Smith JC (1999) Models of respiratory rhythm generation in the pre-botzinger complex. i. bursting pacemaker neurons. J Neurophysiol 82(1):382–397 10400966 10.1152/jn.1999.82.1.382

[CR14] Bolser DC, Pitts TE, Davenport PW, Morris KF (2015) Role of the dorsal medulla in the neurogenesis of airway protection. Pulmonary Pharmacol Ther 35:105–110. 10.1016/j.pupt.2015.10.006 26549786 PMC4690802

[CR15] Bolser DC, Poliacek I, Jakus J, Fuller DD, Davenport PW (2006) Neurogenesis of cough, other airway defensive behaviors and breathing: a holarchical system? Respiratory Physiol Neurobiol 152(3):255–265 10.1016/j.resp.2006.01.008 PMC3121153 16723284

[CR16] Bautista TG, Sun Q-J, Pilowsky PM (2014) The generation of pharyngeal phase of swallow and its coordination with breathing: interaction between the swallow and respiratory central pattern generators. In: Feldman JL, Del Negro CA, McCrimmon DR (eds) Progress brain research, vol 212. Elsevier, Amsterdam, pp 253–275. 10.1016/B978-0-444-63488-7.00012-7 25194202

[CR17] Champagnat J, Jacquin T, Richter D (1986) Voltage-dependent currents in neurones of the nuclei of the solitary tract of rat brainstem slices. Pflügers Archiv Eur J Physiol 406(4):372–379 2423952 10.1007/BF00590939

[CR18] Dhingra RR, Dick TE, Furuya WI, Galán RF, Dutschmann M (2020) Volumetric mapping of the functional neuroanatomy of the respiratory network in the perfused brainstem preparation of rats. J Physiol 598(11):2061–2079 32100293 10.1113/JP279605

[CR19] Daniels SK, Foundas AL (1997) The role of the insular cortex in dysphagia. Dysphagia 12(3):146–156 9190100 10.1007/PL00009529

[CR20] Dhingra RR, Furuya WI, Bautista TG, Dick TE, Galán RF, Dutschmann M (2019a) Increasing local excitability of brainstem respiratory nuclei reveals a distributed network underlying respiratory motor pattern formation. Front Physiol 10:887 31396094 10.3389/fphys.2019.00887 PMC6664290

[CR21] Dhingra RR, Furuya WI, Galán RF, Dutschmann M (2019) Excitation-inhibition balance regulates the patterning of spinal and cranial inspiratory motor outputs in rats in situ. Respiratory Physiol Neurobiol 266:95–102 10.1016/j.resp.2019.05.001 31055189

[CR22] Dutschmann M, Herbert H (2006) The kölliker-fuse nucleus gates the postinspiratory phase of the respiratory cycle to control inspiratory off-switch and upper airway resistance in rat. Eur J Neurosci 24(4):1071–1084 16930433 10.1111/j.1460-9568.2006.04981.x

[CR23] Dutschmann M, Jones SE, Subramanian HH, Stanic D, Bautista TG (2014) The physiological significance of postinspiration in respiratory control. Prog Brain Res 212:113–130 25194196 10.1016/B978-0-444-63488-7.00007-0

[CR24] Dutschmann M, Menuet C, Stettner GM, Gestreau C, Borghgraef P, Devijver H, Gielis L, Hilaire G, Van Leuven F (2010) Upper airway dysfunction of tau-p301l mice correlates with tauopathy in midbrain and ponto-medullary brainstem nuclei. J Neurosci 30(5):1810–1821 20130190 10.1523/JNEUROSCI.5261-09.2010 PMC6633985

[CR25] Del Negro CA, Funk GD, Feldman JL (2018) Breathing matters. Nat Rev Neurosci 19(6):351–367 29740175 10.1038/s41583-018-0003-6 PMC6636643

[CR26] Dutschmann M, Paton JFR (2002) Glycinergic inhibition is essential for co-ordinating cranial and spinal respiratory motor outputs in the neonatal rat. J Physiol 543(2):643–653. 10.1113/jphysiol.2002.019547 12205196 PMC2290509

[CR27] Dutschmann M, Paton JFR (2002) Influence of nasotrigeminal afferents on medullary respiratory neurones and upper airway patency in the rat. Pflugers Arch 444(1):227–235. 10.1007/s004240100645 11976936

[CR28] Dutschmann M, Paton JF (2002) Inhibitory synaptic mechanisms regulating upper airway patency. Respiratory Physiol Neurobiol 131(1–2):57–63 10.1016/s1569-9048(02)00037-x 12106995

[CR29] Dutschmann M, Wilson RJ, Paton JF (2000) Respiratory activity in neonatal rats. Auton Neurosci 84(1–2):19–29 11109986 10.1016/S1566-0702(00)00177-6

[CR30] Ezure K, Oku Y, Tanaka I (1993) Location and axonal projection of one type of swallowing interneurons in cat medulla. Brain Res 632(1–2):216–224 8149230 10.1016/0006-8993(93)91156-m

[CR31] Easterling CS, Robbins E (2008) Dementia and dysphagia. Geriatr Nurs 29(4):275–285 18694703 10.1016/j.gerinurse.2007.10.015

[CR32] Fuse S, Sugiyama Y, Dhingra R, Hirano S, Dutschmann M, Oku Y (2019) Effects of pharmacological lesion of the nucleus retroambiguus region on the pharyngeal phase of swallowing. Respiratory Physiol Neurobiol 268:103244 10.1016/j.resp.2019.06.001 31226424

[CR33] Fuse S, Sugiyama Y, Dhingra RR, Hirano S, Dutschmann M, Okada Y, Oku Y (2025) Spatio-temporal segregation between sensory relay and swallowing pre-motor population activities by optical imaging in the rat nucleus of the solitary tract. Pflügers Archiv-Eur J Physiol 477(5):719–727 39862246 10.1007/s00424-025-03065-9 PMC12003619

[CR34] Finiels H, Strubel D, Jacquot J (2001) Deglutition disorders in the elderly. epidemiological aspects. Presse Med (Paris France 1983) 30(33):1623–1634 11759342

[CR35] Gross RD, Atwood CW Jr, Ross SB, Olszewski JW, Eichhorn KA (2009) The coordination of breathing and swallowing in chronic obstructive pulmonary disease. Am J Respir Crit Care Med 179(7):559–565 19151193 10.1164/rccm.200807-1139OC

[CR36] Gross RD, Atwood CW, Ross SB, Eichhorn KA, Olszewski JW, Doyle PJ (2008) The coordination of breathing and swallowing in parkinson’s disease. Dysphagia 23(2):136–145 18027027 10.1007/s00455-007-9113-4

[CR37] Galán RF, Ermentrout GB, Urban NN (2005) Efficient estimation of phase-resetting curves in real neurons and its significance for neural-network modeling. Phys Rev Lett 94(15):158101 15904191 10.1103/PhysRevLett.94.158101 PMC1410718

[CR38] Guerrier C, Hayes JA, Fortin G, Holcman D (2015) Robust network oscillations during mammalian respiratory rhythm generation driven by synaptic dynamics. Proc Natl Acad Sci 112(31):9728–9733 26195782 10.1073/pnas.1421997112 PMC4534249

[CR39] Golowasch J (2014) Ionic current variability and functional stability in the nervous system. Bioscience 64(7):570–580 26069342 10.1093/biosci/biu070 PMC4460997

[CR40] Goaillard J-M, Taylor AL, Schulz DJ, Marder E (2009) Functional consequences of animal-to-animal variation in circuit parameters. Nat Neurosci 12(11):1424–1430 19838180 10.1038/nn.2404 PMC2826985

[CR41] Harding R (1984) Perinatal development of laryngeal function. J Dev Physiol 6(3):249–258 6747226

[CR42] Harris KD, Dashevskiy T, Mendoza J, Garcia AJ III, Ramirez J-M, Shea-Brown E (2017) Different roles for inhibition in the rhythm-generating respiratory network. J Neurophysiol 118(4):2070–2088 28615332 10.1152/jn.00174.2017 PMC5626906

[CR43] Huff A, Karlen-Amarante M, Oliveira LM, Ramirez J-M (2023) Role of the postinspiratory complex in regulating swallow-breathing coordination and other laryngeal behaviors. Elife 12:86103 10.7554/eLife.86103 PMC10264072 37272425

[CR44] Hilaire G, Monteau R, Gauthier P, Rega P, Morin D (1990) Functional significance of the dorsal respiratory group in adult and newborn rats: in vivo and in vitro studies. Neurosci Lett 111(1–2):133–138 2336178 10.1016/0304-3940(90)90357-f

[CR45] Hooper SL (2001) Central pattern generators. In: Encyclopedia of life sciences (eLS). John Wiley & Sons, Ltd, Chichester. 10.1038/npg.els.0000064

[CR46] Hashimoto K, Sugiyama Y, Fuse S, Umezaki T, Oku Y, Dutschmann M, Hirano S (2019) Activity of swallowing-related neurons in the medulla in the perfused brainstem preparation in rats. Laryngoscope 129(2):72–79 10.1002/lary.27401 30408193

[CR47] Horton K-K, Segers LS, Nuding SC, O’Connor R, Alencar PA, Davenport PW, Bolser DC, Pitts T, Lindsey BG, Morris KF et al (2018) Central respiration and mechanical ventilation in the gating of swallow with breathing. Front Physiol 9:785 30013484 10.3389/fphys.2018.00785 PMC6036260

[CR48] Hiss SG, Strauss M, Treole K, Stuart A, Boutilier S (2003) Swallowing apnea as a function of airway closure. Dysphagia 18(4):293–300 14571335 10.1007/s00455-003-0021-y

[CR49] Izhikevich EM (2007) Dynamical systems in neuroscience: the geometry of excitability and bursting. MIT Press, Cambridge, MA. 10.7551/mitpress/2526.001.0001

[CR50] Jean A CA (1979) Inputs to the swallowing medullary neurons from the peripheral afferent fibers and the swallowing cortical area. Brain Res 178(2–3):567–572 509218 10.1016/0006-8993(79)90715-7

[CR51] Jean A, Dallaporta M (2006) Electrophysiologic characterization of the swallowing pattern generator in the brainstem. GI Motility Online. 10.1038/gimo9

[CR52] Jean A (2001) Brain stem control of swallowing: neuronal network and cellular mechanisms. Physiol Rev 81(2):929–969 11274347 10.1152/physrev.2001.81.2.929

[CR53] Jafari S, Prince RA, Kim DY, Paydarfar D (2003) Sensory regulation of swallowing and airway protection: a role for the internal superior laryngeal nerve in humans. J Physiol 550(1):287–304 12754311 10.1113/jphysiol.2003.039966 PMC2343009

[CR54] Kralemann B, Cimponeriu L, Rosenblum M, Pikovsky A, Mrowka R (2008) Phase dynamics of coupled oscillators reconstructed from data. Phys Rev E 77(6):066205 10.1103/PhysRevE.77.066205 18643348

[CR55] Kessler J, Jean A (1985) Identification of the medullary swallowing regions in the rat. Exp Brain Res 57(2):256–263 3972029 10.1007/BF00236530

[CR56] Kalia M, Mesulam M-M (1980) Brain stem projections of sensory and motor components of the vagus complex in the cat: Ii. laryngeal, tracheobronchial, pulmonary, cardiac, and gastrointestinal branches. J Comparative Neurol 193(2):467–508 10.1002/cne.901930211 7440778

[CR57] Kinoshita S, Sugiyama Y, Hashimoto K, Fuse S, Mukudai S, Umezaki T, Dutschmann M, Hirano S (2021) Influences of gabaergic inhibition in the dorsal medulla on contralateral swallowing neurons in rats. Laryngoscope 131(10):2187–2198 33146426 10.1002/lary.29242

[CR58] Lang IM (2009) Brain stem control of the phases of swallowing. Dysphagia 24(3):333–348 19399555 10.1007/s00455-009-9211-6

[CR59] Lewis J, Bachoo M, Polosa C, Glass L (1990) The effects of superior laryngeal nerve stimulation on the respiratory rhythm: phase-resetting and aftereffects. Brain Res 517(1–2):44–50 2376006 10.1016/0006-8993(90)91005-2

[CR60] Leslie P, Drinnan MJ, Ford GA, Wilson JA (2002) Swallow respiration patterns in dysphagic patients following acute stroke. Dysphagia 17(3):202–207 12140646 10.1007/s00455-002-0053-8

[CR61] Lindsey BG, Rybak IA, Smith JC (2012) Computational models and emergent properties of respiratory neural networks. Compr Physiol 2(3):1619–1670 23687564 10.1002/cphy.c110016 PMC3656479

[CR62] Ludlow CL (2015) Central nervous system control of voice and swallowing. J Clin Neurophysiol 32(4):294–303 26241238 10.1097/WNP.0000000000000186 PMC4526113

[CR63] Matsuoka K (2011) Analysis of a neural oscillator. Biol Cybern 104(4–5):297–304. 10.1007/s00422-011-0432-z 21562853

[CR64] Mosier K, Bereznaya I (2001) Parallel cortical networks for volitional control of swallowing in humans. Exp Brain Res 140(3):280–289 11681303 10.1007/s002210100813

[CR65] Marder E, Bucher D (2007) Understanding circuit dynamics using the stomatogastric nervous system of lobsters and crabs. Annu Rev Physiol 69:291–316 17009928 10.1146/annurev.physiol.69.031905.161516

[CR66] Molkov YI, Bacak BJ, Dick TE, Rybak IA (2013) Control of breathing by interacting pontine and pulmonary feedback loops. Front Neural Circuits 7:16 23408512 10.3389/fncir.2013.00016 PMC3570896

[CR67] Molkov Y, Borgmann A, Koizumi H, Hama N, Zhang R, Smith J (2025) Inference technique for the synaptic conductances in rhythmically active networks and application to respiratory central pattern generation circuits. Elife 13:101959 10.7554/eLife.101959 PMC12221296 40600810

[CR68] Menuet C, Borghgraef P, Matarazzo V, Gielis L, Lajard A-M, Voituron N, Gestreau C, Dutschmann M, Van Leuven F, Hilaire G (2011) Raphe tauopathy alters serotonin metabolism and breathing activity in terminal tau. p301l mice: possible implications for tauopathies and alzheimer’s disease. Respiratory Physiol Neurobiol 178(2):290–303 10.1016/j.resp.2011.06.030 21763469

[CR69] McCarty EB, Chao TN (2021) Dysphagia and swallowing disorders. Medical. Clinics 105(5):939–954 10.1016/j.mcna.2021.05.013 34391544

[CR70] Marder E, Goeritz ML, Otopalik AG (2015) Robust circuit rhythms in small circuits arise from variable circuit components and mechanisms. Curr Opin Neurobiol 31:156–163 25460072 10.1016/j.conb.2014.10.012 PMC4375070

[CR71] Miller AJ (1986) Neurophysiological basis of swallowing. Dysphagia 1(2):91–100

[CR72] Molkov YI, Koizumi H, Smith JC (2026) Functional synaptic interactions and inhibitory circuitry of the prebötzinger complex in the rhythmic slice. bioRxiv, 2026–0510.1152/jn.00235.202642537403

[CR73] Miller AJ, Loizzi RF (1974) Anatomical and functional differentiation of superior laryngeal nerve fibers affecting swallowing and respiration. Exp Neurol 42(2):369–387 4824983 10.1016/0014-4886(74)90033-8

[CR74] Molineux ML, Mehaffey WH, Tadayonnejad R, Anderson D, Tennent AF, Turner RW (2008) Ionic factors governing rebound burst phenotype in rat deep cerebellar neurons. J Neurophysiol 100(5):2684–2701 18768644 10.1152/jn.90427.2008

[CR75] Molkov YI, Rubin JE, Rybak IA, Smith JC (2017) Computational models of the neural control of breathing. Wiley Interdiscipl Rev Sys Biol Medi 9(2):1371 10.1002/wsbm.1371 PMC5315581 28009109

[CR76] Molkov YI, Shevtsova NA, Park C, Ben-Tal A, Smith JC, Rubin JE, Rybak IA (2014) A closed-loop model of the respiratory system: focus on hypercapnia and active expiration. PLoS ONE 9(10):109894 10.1371/journal.pone.0109894 PMC4193835 25302708

[CR77] Maynard TM, Zohn IE, Moody SA, LaMantia A-S (2020) Suckling, feeding, and swallowing: behaviors, circuits, and targets for neurodevelopmental pathology. Annu Rev Neurosci 43(1):315–336 32101484 10.1146/annurev-neuro-100419-100636 PMC7359496

[CR78] Niederman MS, Cilloniz C (2022) Aspiration pneumonia Revista Espanola de Quimioterapia 35(Suppl 1):73 35488832 10.37201/req/s01.17.2022 PMC9106188

[CR79] Nagornov R, Osipov G, Komarov M, Pikovsky A, Shilnikov A (2016) Mixed-mode synchronization between two inhibitory neurons with post-inhibitory rebound. Commun Nonlinear Sci Numer Simul 36:175–191

[CR80] Nagami S, Oku Y, Yagi N, Sato S, Uozumi R, Morita S, Yamagata Y, Kayashita J, Tanimura K, Sato A et al (2017) Breathing-swallowing discoordination is associated with frequent exacerbations of copd. BMJ Open Respir Res 4(1):000202 10.1136/bmjresp-2017-000202 PMC5531308 28883930

[CR81] Oku Y, Dick TE (1992) Phase resetting of the respiratory cycle before and after unilateral pontine lesion in cat. J Appl Physiol 72(2):721–730 1559953 10.1152/jappl.1992.72.2.721

[CR82] Paton JF (1996) A working heart-brainstem preparation of the mouse. J Neurosci Methods 65(1):63–68 8815310 10.1016/0165-0270(95)00147-6

[CR83] Pitts T, Bolser D, Rosenbek J, Troche M, Sapienza C (2008) Voluntary cough production and swallow dysfunction in parkinson’s disease. Dysphagia 23(3):297–301 18483823 10.1007/s00455-007-9144-x PMC4080193

[CR84] Pitts T, Huff A, Reed M, Iceman K, Mellen N (2021) Evidence of intermediate reticular formation involvement in swallow pattern generation, recorded optically in the neonate rat sagittally-sectioned hindbrain. J Neurophysiol 125(3):890–907. 10.1152/jn.00519.2020 PMC8282227 33566745

[CR85] Pitts T, Iceman KE (2023) Deglutition and the regulation of the swallow motor pattern. Physiology 38(1):10–24 10.1152/physiol.00005.2021 PMC9707372 35998250

[CR86] Paton JF, Machado BH, Moraes DJ, Zoccal DB, Abdala AP, Smith JC, Antunes VR, Murphy D, Dutschmann M, Dhingra RR et al (2022) Advancing respiratory-cardiovascular physiology with the working heart-brainstem preparation over 25 years. J Physiol 600(9):2049–2075 35294064 10.1113/JP281953 PMC9322470

[CR87] Pitts T, Rose MJ, Mortensen AN, Poliacek I, Sapienza CM, Lindsey BG, Morris KF, Davenport PW, Bolser DC (2013) Coordination of cough and swallow: a meta-behavioral response to aspiration. Respiratory Physiol Neurobiol 189(3):543–551 10.1016/j.resp.2013.08.009 PMC3882902 23998999

[CR88] Potts JT, Rybak IA, Paton JF (2005) Respiratory rhythm entrainment by somatic afferent stimulation. J Neurosci 25(8):1965–1978 15728836 10.1523/JNEUROSCI.3881-04.2005 PMC6726065

[CR89] Ptak K, Zummo GG, Alheid GF, Tkatch T, Surmeier DJ, McCrimmon DR (2005) Sodium currents in medullary neurons isolated from the pre-bötzinger complex region. J Neurosci 25(21):5159–5170 15917456 10.1523/JNEUROSCI.4238-04.2005 PMC6724824

[CR90] Ramirez J-M, Baertsch N (2018) Defining the rhythmogenic elements of mammalian breathing. Physiology 33(5):302–316 30109823 10.1152/physiol.00025.2018 PMC6230551

[CR91] Rüb U, Del Tredici K, Schultz C, Thal D, Braak E, Braak H (2001) The autonomic higher order processing nuclei of the lower brain stem are among the early targets of the alzheimer’s disease-related cytoskeletal pathology. Acta Neuropathol 101(6):555–564 11515783 10.1007/s004010000320

[CR92] Rybak IA, Paton JF, Schwaber JS (1997) Modeling neural mechanisms for genesis of respiratory rhythm and pattern. i. models of respiratory neurons. J Neurophysiol 77(4):1994–2006 9114250 10.1152/jn.1997.77.4.1994

[CR93] Rubin JE, Smith JC (2019) Robustness of respiratory rhythm generation across dynamic regimes. PLoS Comput Biol 15(7):1006860 10.1371/journal.pcbi.1006860 PMC6697358 31361738

[CR94] Rubin JE, Shevtsova NA, Ermentrout GB, Smith JC, Rybak IA (2009) Multiple rhythmic states in a model of the respiratory central pattern generator. J Neurophysiol 101(4):2146–2165 19193773 10.1152/jn.90958.2008 PMC2695631

[CR95] Rybak I, Shevtsova N, Paton J, Dick T, John WS, Mörschel M, Dutschmann M (2004) Modeling the ponto-medullary respiratory network. Respiratory Physiol Neurobiol 143(2–3):307–319 10.1016/j.resp.2004.03.020 15519563

[CR96] Rybak IA, Shevtsova NA, Paton JFR, Pierrefiche O, St-John WM, Haji A (2004) Modelling respiratory rhythmogenesis: Focus on phase switching mechanisms. In: Champagnat J, Fortin G, Simmers J, Viana Di Prisco G, Milsom WK (eds.) Post-Genomic Perspectives in Modeling and Control of Breathing. Advances in Experimental Medicine and Biology, vol 551. Springer, Boston, MA, pp 189–194. 10.1007/978-1-4419-8896-8_23 15602963

[CR97] Seçil Y, Arıcı Ş, İncesu TK, Gürgör N, Beckmann Y, Ertekin C (2016) Dysphagia in alzheimer’s disease. Neurophysiologie Clinique/Clinical Neurophysiol 46(3):171–178 10.1016/j.neucli.2015.12.007 26924307

[CR98] Smith JC, Abdala A, Koizumi H, Rybak IA, Paton JF (2007) Spatial and functional architecture of the mammalian brain stem respiratory network: a hierarchy of three oscillatory mechanisms. J Neurophysiol 98(6):3370–3387 17913982 10.1152/jn.00985.2007 PMC2225347

[CR99] Sun Q-J, Bautista TG, Berkowitz RG, Zhao W-J, Pilowsky PM (2011) The temporal relationship between non-respiratory burst activity of expiratory laryngeal motoneurons and phrenic apnoea during stimulation of the superior laryngeal nerve in rat. J Physiol 589(7):1819–1830 21320890 10.1113/jphysiol.2010.203794 PMC3099032

[CR100] Sato K, Chitose S-I, Sato K, Umeno H (2016) Deglutition and respiratory patterns during sleep in the aged. Acta Otolaryngol 136(12):1278–1284 27400151 10.1080/00016489.2016.1203991

[CR101] Smith JC, Ellenberger HH, Ballanyi K, Richter DW, Feldman JL (1991) Pre-botzinger complex: a brainstem region that may generate respiratory rhythm in mammals. Science 254(5032):726–729 1683005 10.1126/science.1683005 PMC3209964

[CR102] Saito Y, Ezure K, Tanaka I (2002) Swallowing-related activities of respiratory and non-respiratory neurons in the nucleus of solitary tract in the rat. J Physiol 540(3):1047–1060 11986389 10.1113/jphysiol.2001.014985 PMC2290262

[CR103] Saito Y, Ezure K, Tanaka I, Osawa M (2003) Activity of neurons in ventrolateral respiratory groups during swallowing in decerebrate rats. Brain Develop 25(5):338–345 10.1016/s0387-7604(03)00008-1 12850513

[CR104] Sang Q, Goyal RK (2001) Swallowing reflex and brain stem neurons activated by superior laryngeal nerve stimulation in the mouse. Am J Physiol-Gastrointestinal Liver Physiol 280(2):191–200 10.1152/ajpgi.2001.280.2.G191 11208540

[CR105] Schulz DJ, Goaillard J-M, Marder E (2006) Variable channel expression in identified single and electrically coupled neurons in different animals. Nat Neurosci 9(3):356–362 16444270 10.1038/nn1639

[CR106] St-John WM, Paton JFR (2003) Defining eupnea. Respiratory Physiol Neurobiol 139(1):97–103 10.1016/s1569-9048(03)00193-9 14637316

[CR107] Siegle JH, López AC, Patel YA, Abramov K, Ohayon S, Voigts J (2017) Open ephys: an open-source, plugin-based platform for multichannel electrophysiology. J Neural Eng 14(4):045003 28169219 10.1088/1741-2552/aa5eea

[CR108] Sejdic E, Malandraki GA, Coyle JL (2019) Computational deglutition: using signal-and image-processing methods to understand swallowing and associated disorders. IEEE Signal Process Mag 36(1):138–146 31631954 10.1109/MSP.2018.2875863 PMC6800740

[CR109] Sugiyama Y, Shiba K, Nakazawa K, Suzuki T, Umezaki T, Ezure K, Abo N, Yoshihara T, Hisa Y (2011) Axonal projections of medullary swallowing neurons in guinea pigs. J Comparative Neurol 519(11):2193–2211 10.1002/cne.22624 21456000

[CR110] Sato K, Umeno H, Chitose S-I, Nakashima T (2011) Sleep-related deglutition in patients with osahs under cpap therapy. Acta Otolaryngol 131(2):181–189 21062120 10.3109/00016489.2010.520166

[CR111] Sullivan PB (2008) Gastrointestinal disorders in children with neurodevelopmental disabilities. Dev Disabil Res Rev 14(2):128–136 10.1002/ddrr.18 18646021

[CR112] Tell F, Bradley RM (1994) Whole-cell analysis of ionic currents underlying the firing pattern of neurons in the gustatory zone of the nucleus tractus solitarii. J Neurophysiol 71(2):479–492 7513751 10.1152/jn.1994.71.2.479

[CR113] Trevizan-Baú P, Stanić D, Furuya WI, Dhingra RR, Dutschmann M (2024) Neuroanatomical frameworks for volitional control of breathing and orofacial behaviors. Respiratory Physiol Neurobiol 323:104227 10.1016/j.resp.2024.104227 38295924

[CR114] Tolmachev P, Dhingra RR, Pauley M, Dutschmann M, Manton JH (2018) Modeling the respiratory central pattern generator with resonate-and-fire izhikevich-neurons. In: International conference on neural information processing. Springer, pp 603–615

[CR115] Tell F, Jean A (1991) Activation of n-methyl-d-aspartate receptors induces endogenous rhythmic bursting activities in nucleus tractus solitarii neurons: An intracellular study on adult rat brainstem slices. Eur J Neurosci 3(12):1353–1365 12106233 10.1111/j.1460-9568.1991.tb00068.x

[CR116] Tell F, Jean A (1993) Ionic basis for endogenous rhythmic patterns induced by activation of n-methyl-d-aspartate receptors in neurons of the rat nucleus tractus solitarii. J Neurophysiol 70(6):2379–2390 7509858 10.1152/jn.1993.70.6.2379

[CR117] Toor RUAS, Sun Q-J, Kumar NN, Le S, Hildreth CM, Phillips JK, McMullan S (2019) Neurons in the intermediate reticular nucleus coordinate postinspiratory activity, swallowing, and respiratory-sympathetic coupling in the rat. J Neurosci 39(49):9757–9766 31666354 10.1523/JNEUROSCI.0502-19.2019 PMC6891060

[CR118] Takemura A, Sugiyama Y, Yamamoto R, Kinoshita S, Kaneko M, Fuse S, Hashimoto K, Mukudai S, Umezaki T, Dutschmann M et al (2022) Effect of pharmacological inhibition of the pontine respiratory group on swallowing interneurons in the dorsal medulla oblongata. Brain Res 1797:148101 36183794 10.1016/j.brainres.2022.148101

[CR119] Virtanen P et al (2020) Scipy 1.0: fundamental algorithms for scientific computing in python. Nat Methods 17:261–272. 10.1038/s41592-019-0686-2 32015543 PMC7056644

[CR120] Wang Y, Rubin JE (2020) Complex bursting dynamics in an embryonic respiratory neuron model. Chaos An Interdiscipl J Nonlinear Sci 30(4):043127 10.1063/1.5138993 32357647

[CR121] Wei K-C, Wang T-G, Hsiao M-Y (2024) The cortical and subcortical neural control of swallowing: a narrative review. Dysphagia 39(2):177–197 37603047 10.1007/s00455-023-10613-x

[CR122] Yagi N, Oku Y, Nagami S, Yamagata Y, Kayashita J, Ishikawa A, Domen K, Takahashi R (2017) Inappropriate timing of swallow in the respiratory cycle causes breathing-swallowing discoordination. Front Physiol 8:676. 10.3389/fphys.2017.00676 28970804 PMC5609438

